# Predicting incident cardio-metabolic disease among persons with and without depressive and anxiety disorders: a machine learning approach

**DOI:** 10.1007/s00127-025-02857-9

**Published:** 2025-02-18

**Authors:** Arja O. Rydin, George Aalbers, Wessel A. van Eeden, Femke Lamers, Yuri Milaneschi, Brenda W. J. H. Penninx

**Affiliations:** 1 Department of Psychiatry, Amsterdam UMC location Vrije Universiteit Amsterdam, Boelelaan, Amsterdam, 1117 The Netherlands; 2 Mental Health Program, Amsterdam Public Health, Amsterdam, The Netherlands; 3 Department of Psychiatry, Leiden University Medical Centre, Leiden University, Leiden, The Netherlands; 4 Amsterdam Neuroscience, Mood, Anxiety, Psychosis, Sleep & Stress program, Amsterdam, The Netherlands

**Keywords:** Machine learning, Cardiometabolic diseases, Depression, Anxiety, Comorbidity

## Abstract

**Purpose:**

There is a global increase of cardiovascular disease and diabetes (Cardio-Metabolic diseases: CMD). Suffering from depression or anxiety disorders increases the probability of developing CMD. In this study we tested a wide array of predictors for the onset of CMD with Machine Learning (ML), evaluating whether adding detailed psychiatric or biological variables increases predictive performance.

**Methods:**

We analysed data from the Netherlands Study of Depression and Anxiety, a longitudinal cohort study (*N* = 2071), using 368 predictors covering 4 domains (demographic, lifestyle & somatic, psychiatric, and biological markers). CMD onset (24% incidence) over a 9-year follow-up was defined using self-reported stroke, heart disease, diabetes with high fasting glucose levels and (antithrombotic, cardiovascular, or diabetes) medication use (ATC codes C01DA, C01-C05A-B, C07-C09A-B, C01DB, B01, A10A-X). Using different ML methods (Logistic regression, Support vector machine, Random forest, and XGBoost) we tested the predictive performance of single domains and domain combinations.

**Results:**

The classifiers performed similarly, therefore the simplest classifier (Logistic regression) was selected. The Area Under the Receiver Operator Characteristic Curve (AUC-ROC) achieved by singe domains ranged from 0.569 to 0.649. The combination of demographics, lifestyle & somatic indicators and psychiatric variables performed best (AUC-ROC = 0.669), but did not significantly outperform demographics. Age and hypertension contributed most to prediction; detailed psychiatric variables added relatively little.

**Conclusion:**

In this longitudinal study, ML classifiers were not able to accurately predict 9-year CMD onset in a sample enriched of subjects with psychopathology. Detailed psychiatric/biological information did not substantially increase predictive performance.

**Supplementary Information:**

The online version contains supplementary material available at 10.1007/s00127-025-02857-9.

## Introduction

The prevalence of cardiovascular diseases and Diabetes Mellitus type II (further referred to as cardiometabolic diseases, or CMD) is increasing and constitutes a prominent cause of death and impairment of life quality world-wide [1–3]. The etiological mechanisms involved in these diseases are complex, but a few known risk factors are age, HDL cholesterol, BMI, triglycerides and blood pressure (also known as the Framingham risk score [4]). It is clear that different domains such as lifestyle, sociodemographic, biological and psychosocial factors are at play [5–8].

In the psychosocial domain, mental health factors such as depression and anxiety, are established risk factors of CMD [9–13]. This could partly be explained by diverse dysregulated biological immunometabolic mechanisms [14] as different sets of -omics data (metabolomics, proteomics, lipidomics) have confirmed poorer biological cardiometabolic health profiles among depressed cases compared to controls [15–18]. However, it has not yet been studied to what extent information on mental health factors might contribute to the prediction of CMD development in addition to known risk factors such as high blood pressure, age and BMI.

There is no single factor that fully explains why some people develop CMD, whereas others do not. Similar to depression, lifestyle factors (e.g. smoking, alcohol use, physical activity), somatic indicators (e.g. BMI, medication use) and biological markers (e.g. inflammation, metabolic syndrome) are also known risk factors for CMD [19–22]. CMD are multifactorial and clusters of risk factors might interact [23]. These factors are not only linked to the development of CMD, they are also associated with depression. This overlap gives rise to the question which factors contribute to the onset of CMD when looking at a population with a higher endorsement of depression and anxiety than the general population.

So far, many prediction studies are either limited in the number of predictive markers they link [24–26], or do not exploit statistical methods that can capture non-linear relations between a large number of features and outcomes. For instance, Chu et al. [27], used Deep Neural Network to predict cardiovascular diseases in a population of people with diabetes, using amongst others measures of depression and anxiety. In their analysis, anxiety was the third leading impact factor for the prediction of CVD. More complex models can capture non-linear relations between a large number of features and outcomes. With the availability of a wide variety of domains collected for instance by medical organisations or observational cohort studies, Machine Learning (ML) tools may help us to optimally analyse such big data. These methods can exploit data and help make predictions on the onset of CMD more adequately than “classical” statistical methods [20, 21, 28]. The collected data is oftentimes multidimensional; variables may have multi-collinearity amongst one another; absolute outcomes may be low: at a first glance it may not be clear which variables are most important. ML methods can tackle that problem, as they allow a large number of predictor variables to be used at once.

This study aimed to examine which factors predict the cumulative onset of CMD in a population with a higher proportion of persons with depression and anxiety diagnoses, thereby focussing on the contribution of mental health factors and biological markers in addition to established risk factors. Four domains of predictors were used: sociodemographic variables, lifestyle & somatic indicators, psychiatric variables, and biological markers. The psychiatric domain contained almost 50 indicators of mental health ranging from self-reported symptoms to clinically assessed diagnoses, making the dataset unique in the depth of the mental health dimension. The biological domain consisted of more than 300 markers, including a full lipidomic and proteomic panel and markers of inflammation. We compared different classifiers selecting the simplest, best-performing classifier; after that step, we compared different domains. We selected the demographic domain as a base predictor set, whereafter the other domains were added to evaluate added predictive performance. We assessed whether the psychiatric and biological dimensions are potential additions to the already robust set of known risk factors for CMD.

## Methods

### Participants

Data came from participants in the Netherlands Study of Depression and Anxiety (NESDA) [29], an ongoing longitudinal cohort study on the long-term consequences and course of depressive and anxiety disorders. At baseline, 2,981 participants were included in the age-range of 18–65, between 2004 and 2007. They were recruited from community (19.0%), primary care (54.0%), and specialised mental health care settings (27.0%). The study protocol was approved by the Medical Ethical Committee of participating institutes and all participants provided written informed consent. We included 2,071 subjects without cardiometabolic disease (CMD) at baseline and data availability of CMD at 2-, 4-, 6-, and/or 9-year follow-up, meaning that each subject needed to have information on the outcome in at least one follow-up wave: 910 subjects were excluded due to having CMD at baseline or having no follow-up data on the outcome indicators.

### Onset of cardiometabolic disease (CMD) during follow-up

The outcome variable was defined by the presence of any one of the following 6 indicators: (i) self-reported heart disease; (ii) self-reported stroke; (iii) self-reported diabetes; (iv) cardiovascular medication (ATC codes: C01DA, C01-C05A-B, C07-C09A-B, C01DB); (v) antithrombotic agents (ATC code: B01); or (vi) fasting glucose levels over 7.0 mmol/L or diabetes medication (ATC codes: A10A-X).

### Predictor domains

A total of 383 predictors were included in the dataset of 2,071 subjects. The predictors fall into 4 different categories, described below. A full list of predictors is given in Supplemental Table S1, including their missingness.

### Sociodemographic predictors

Eight sociodemographic variables were included: sex, age, level of education (highest attained degree), birth country, number of nationalities, first nationality, and ancestry of the respondent.

### Lifestyle and somatic health predictors

We selected 15 variables in the lifestyle and somatic health domain, including measures for physical activity, smoking and drinking behaviours, but also BMI, hand grip strength and abdominal obesity.

### Psychiatric predictors

The psychiatric domain consisted of 49 variables, ranging from information on depression, anxiety disorder, antidepressant use and self-reported personality to childhood trauma. For depression symptoms, we used the 30 individual depressive symptoms from the self-report Inventory for Depressive Symptomatology (IDS) [30], because some individual symptoms are more strongly linked to CMD outcomes than others (e.g. physical symptoms such as loss of energy or increased appetite) [17]. Each symptom in the IDS is represented by 4 statements on an ordinal 0–3 scale (‘0=’I do not feel sad’, 3=’I feel almost always feel sad’). We also included the IDS sum score, a one-dimensional scale of depression severity (range 0–84). Furthermore, several depressive and anxiety disorder diagnoses were included as predictors (diagnosis in the last 6 months of dysthymia, major depressive disorder (MDD), social phobia, panic with and without agoraphobia, generalised anxiety disorder, and number of MDD episodes). These were determined using the Composite Interview Diagnostic Instrument (CIDI)– lifetime version 2.1 [31]. which assesses diagnostic criteria of the Diagnostic and Statistical Manual of Mental Disorders (DSM)-IV. We included antidepressant use (tricyclic, ATC N06AA; selective serotonin reuptake inhibitors, ATC 06AB; and other antidepressants, N06AF) based on drug container inspection. Finally, the NEO-FFI questionnaire assessed five personality traits (neuroticism, extraversion, openness to experience, conscientiousness, and agreeableness) [32], and the childhood trauma questionnaire (providing an index of 0–8) [33] was included to indicate exposure to childhood physical, emotional or sexual abuse before age 16.

### Biological variables

The domain of biological predictors consisted of 311 markers collected through blood plasma samples of the participants at baseline after an overnight fast. (full list in Table S1). The plasma samples were stored in ethylendiaminetetraacetic acid (EDTA) detergent, at a temperature of -80 °C until they were assayed. Different assays were employed. This domain can be divided into three categories, described further below.

#### Lipidomics

Lipids and metabolites came from a lipidomic panel by Nightingale Ltd., measuring 230 metabolites. Following previous studies [34], in the present analyses we focused on the subclass of 51 lipids, fatty acids, and low-molecular-weight metabolites. Data were transformed as per the manufacturer’s recommended procedure: a value of 1 was added to each value, and the natural log transformation was applied and when a value deviated > 5SDs from the mean, they were set as missing (resulting in the removal of 80 subjects) [35]. The samples were shipped in two batches, referred to as batch 1 and batch 2 respectively. After using K-nearest neighbours (KNN) to impute missing values, we corrected for batch-sensitive lipids following previous research [16, 34].

#### Proteomics

Blood plasma samples were assayed with the Myriad RBM DiscoveryMAP 250 + in a Clinical Laboratory Improvement Amendments-certified facility (Myriad RBM; located in Austin, Texas, USA). This assay evaluates 243 analytes involved in several hormonal, immunological, and metabolic pathways utilising multiplexed microbead immunoassays to assess the serum [29]. Proteomics data were collected for a subset of healthy controls and a subset of subjects with an MDD diagnosis [16].

#### Inflammatory markers and other biomarkers

Inflammatory markers such as C-Reactive Protein, Interleukin-6 and number of leucocytes were included [36]. Other biological variables were Brain Derived neurotrophic factor (BDNF) [37], leptin [38] and fasting glucose levels [39]. This category consisted of 16 markers (for distribution properties, see Table S2).

### Analysis

For data handling and the pipeline of the ML analysis we used the programming language Python (version 3.9). Model comparison and data visualisation were done in R (version 4.3.2).

### Data handling

In the demographic domain, lifestyle & somatic indicators and psychiatric domain, there were no or very few missing observations at baseline. In the biological domain, however, more data was missing due to levels being below detection rate; these were imputed with either the minimum or maximum value. Some were due to study design (i.e. proteomics were collected for a subset). These missing values were imputed with KNN using the full list of features. In the metabolites domain we corrected for batch-sensitivity, and we imputed with KNN before correcting for the sensitivity, using only metabolite data. Missing percentages are available in Table S1.

### Machine learning analysis

#### Classifiers and pipeline

We included 4 classifiers: Logistic Regression (LR), Support Vector Machine (SVM), Random Forest (RF) and XGBoost (XGB). These models are simple to implement, and offer insights in both linear (LR, SVM) and non-linear (RF, XGB) relations between features and outcome. Furthermore they are able to handle a large number of predictors. For this reason, these classifiers are often selected in classification tasks.

LR computes the log-odds of a discrete event taking place, given input variable(s). It gives weight(s) to the input variable(s) to calculate the probability of an even taking place. The classifier is linear because it sums the weights. The second classifier, SVM, is also linear; it assumes variables are p-dimensional points, and points of different classes can be separated by a (p-1)-dimensional (hyper-)plane. Opposed to LR and SVM, RF is a non-linear classifier, which combines decision trees with a majority voting procedure. Decision trees are prone to overfitting, but are able to capture non-linear relations between input and outcome. XGBoost is also an ensemble-learning method, similar to RF. It combines ‘weak learners’ (in RF, the individual trees are weak learners), but uses a formal function (using gradient descent) to make the combined learner stronger.

The classifiers were trained on the 4 different domains separately, and on the complete predictor set. This resulted in 20 models. For all models we computed several metrics based on the confusion matrix: AUC ROC, sensitivity, specificity, accuracy, balanced accuracy, F1-score, and precision. The AUC ROC curves were also computed (See supplemental materials for Figures S1-S20).

The dataset had a train-test ratio of 80 − 20. First, all predictors in the training set were scaled using the MinMaxScaler function. We trained the classifiers on the 4 separate domains, and on the complete predictor set. When a predictor domain had more than 15 predictors, we used Mutual Information to determine which features had the highest predictive power in order to reduce overfitting, and selected those. Then, the classifier was trained with stratified 10-fold cross-validation to maximise the AUC ROC. We performed an optimisation step by defining a finer search space based on the selected hyperparameters of the randomised search, followed by a grid search procedure. The grid search space parameter values can be found in Table S3. For the randomised search space parameters we refer to Tables S4-S7. To determine which classifier performed best, we did a pair-wise comparison of the AUC ROC of the classifiers values using DeLong’s test [40] within each domain, similar to previous work [41]. We then performed a pair-wise comparison of the AUC ROC values of the domains, again using DeLong’s test. In the case of a tie between classifiers, we chose the classifier with highest explainability and lowest complexity. The ordering was LR, RF, XGB, and SVM, respectively.

After selecting the classifier, we analysed the data through the following steps: (i) we examined the prediction of CMD onset achieved by the single domains; (ii) we tested the incremental AUC ROCs in CMD onset prediction obtained by adding combinations of domains to the benchmark sociodemographic domain; (iii) we examined the overall prediction performance in terms of the AUC ROCs and other evaluation metrics achieved by the full model (all 4 domains combined); (iv) we analysed the importance of individual features in the prediction of CMD onset achieved by the full model by making use of Shapley values [42].

## Results

### Descriptives

The mean age of the sample was 40 years, 68% was female; for these and other characteristics of the data we refer to Table 1. Out of 2,071 subjects, 501 (24.1%) reported a positive outcome for CMD after baseline assessment at FU2, FU4, FU6 or FU9. Of these 501 subjects, 215 (42.9%) reported positive on exactly 1 outcome indicator (e.g. self-reported heart disease), 207 (41.3%) reported positive on 2 outcome indicators, and 79 (15.8%) reported positive for 3 or more indicators. Some pairs or triples of outcome indicators showed up more often than others (Table S8). For instance the combination heart disease and antithrombotic medication, or the combination heart disease, antithrombotic medication, and cardiovascular medication were both relatively prevalent). This overlap of indicators was to be expected, as for instance people with heart disease are prescribed antithrombotic agents and/or cardiovascular medication, and people with diabetes receive insulin. The heatmap (Figure S21) shows the overlap of the 6 indicators and the overall disease outcome.

**Table 1:** Sample characteristics

Sample (*N* = 2071)		
Female (%)		68.8
Mean age (std)		40 (12.8)
Educational attainment (%)	Basic	9.6
	Intermediate	83.8
	High	50.5
MDD diagnosis (%)		35.7
Social phobia (%)		21.5
Panic disorder (%)		20.6
Generalised anxiety disorder (%)		14
Mean BMI (std)		25 (4.8)
Smoking status (%)	Never	30.4
	Former	31.6
	Current	38.0
Alcohol use, days per week (std)		3.2 (1.5)
Physical activity average score (std)		2.1 (0.7)
Presence of CMD indicators (%)†	Heart disease	4.7
	Diabetes	1.6
	Stroke	1.6
	Diabetes medication/high glucose	2.8
	Antithrombotic medication	12.0
	Cardiovascular medication	20.5
Incidence CMD (%)		24.1
Incidence 1 outcome indicator (%)		42.9
Incidence 2 outcome indicators (%)		41.3
Incidence > 3 outcome indicators (%)		15.8

Abbreviations– MDD: Major depressive disorder, BMI: Body mass index, CMD: Cardio-metabolic disease

† Note: subjects can be positive on one or more of these indicators

### Model evaluation

**Fig. 1:**
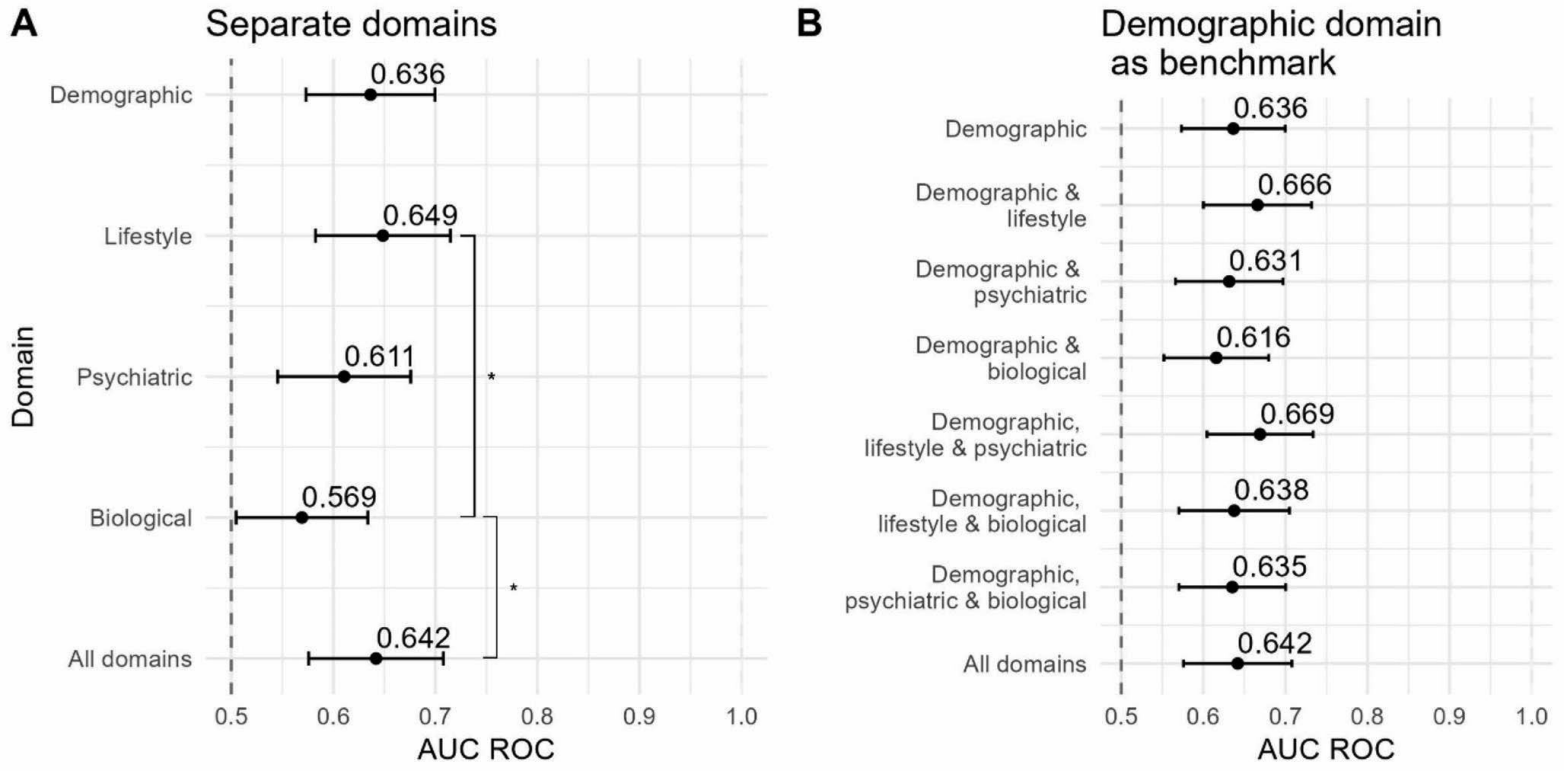
Forest plot with AUC ROC values

We compared four classifiers. There was no significant difference in performance across the four different classifiers, and we thus highlight LR because it is the classifier with lowest complexity and highest explainability. Figure 1A shows the first step of the analysis: the AUC ROCs of the classifier trained on the separate domains. The benchmark domain, demographics, had an AUC ROC of 0.636. The lifestyle domain reached the highest AUC ROC (0.649); the biological domain had the lowest AUC ROC (0.569). In Fig. 1B we see the second step: the incremental performances of the models when adding 1, 2, or 3 domains to the benchmark set. The addition of the lifestyle domain to the benchmark increased the AUC ROC marginally (0.666); adding other single domains to the benchmark decreased the AUC ROC, albeit marginally. The highest AUC ROC is achieved by demographics, lifestyle & somatic and psychiatric variables (AUC ROC = 0.669), but is not significantly higher than other feature set combinations. Combining all domains resulted in an AUC ROC = 0.642.

**Fig. 2:**
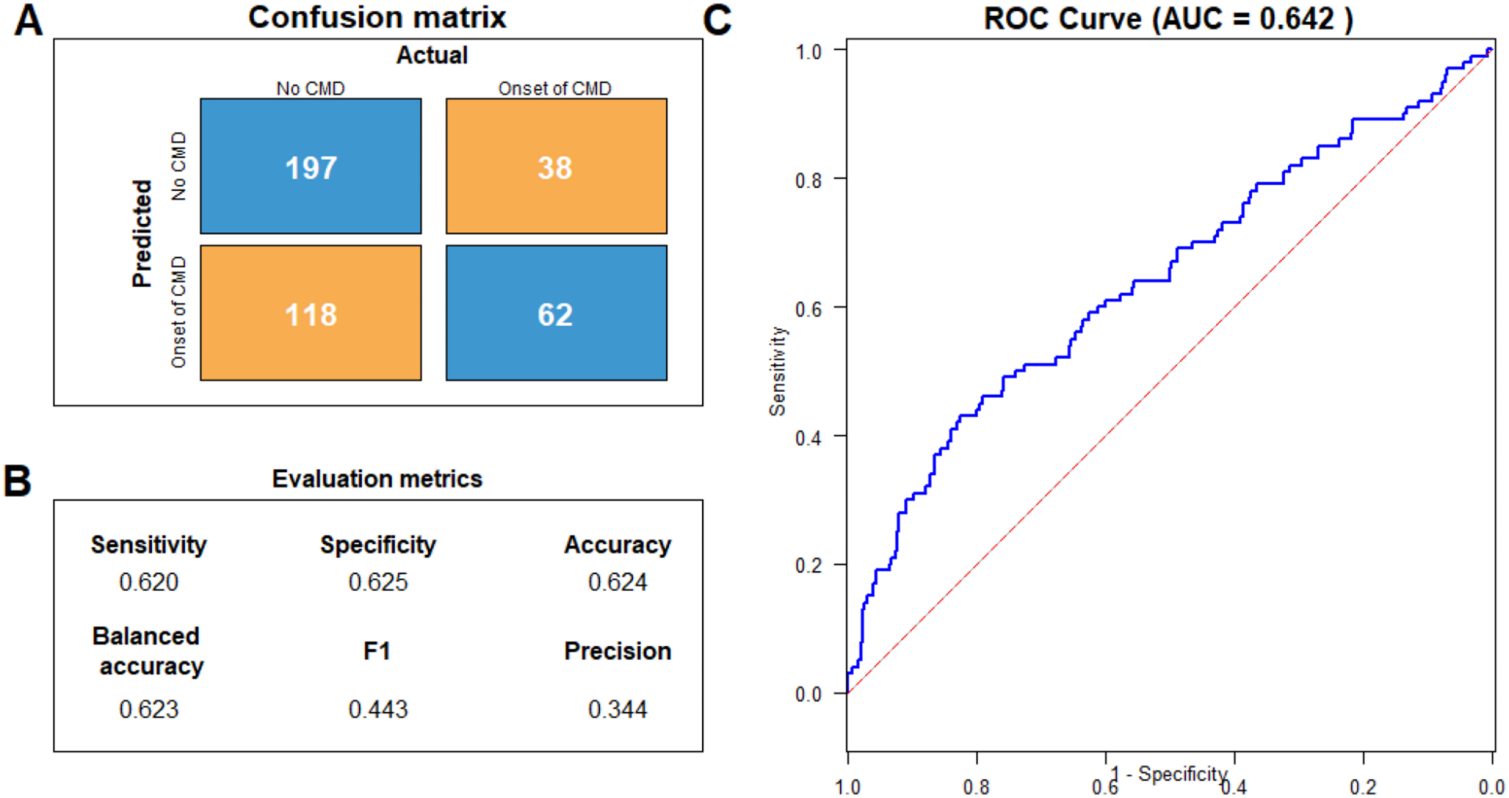
Model performance of the logistic regression trained on the full model containing all domains. **A**. Confusion matrix showing the predicted onsets and actual onsets of CMD; **B**. Evaluation metrics with sensitivity, specificity, accuracy, balanced accuracy, F1 score and precision; **C**. ROC curve belonging to the model

We examined and highlighted the full model’s performances by showing the confusion matrix (Fig. 2A) for the third step (the full model). The model achieved a sensitivity and specificity of 0.620 and 0.625 respectively. The AUC ROC was 0.642, accuracy and balanced accuracies were 0.624 and 0.623. F1-score was 0.443 and precision 0.344 (Fig. 2B). Figure 2C shows the ROC curve.

### Feature importance

**Fig. 3:**
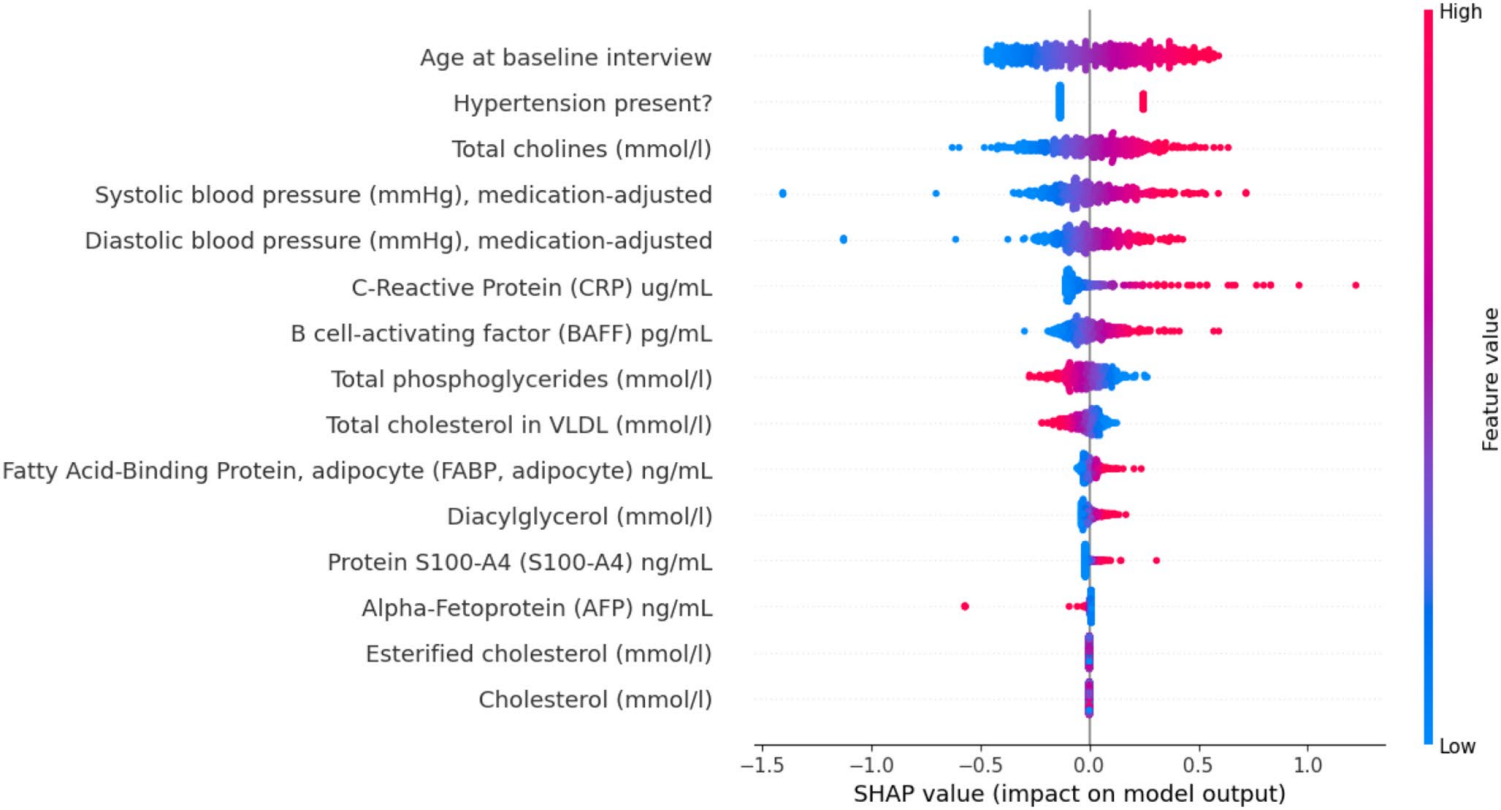
Beeswarm plot of the SHAP values signifying the importance of each variable on the model’s prediction

The fourth and last step was the feature importance analysis with a beeswarm plot, using Shapley Additive explanations (SHAP). A SHAP beeswarm plot visualizes the impact of each feature on the output of a ML model, ranking features by their importance. Each point represents a SHAP value for an individual instance, with the position along the x-axis indicating the magnitude and direction of a feature’s contribution. Point coloured by feature (e.g., from low to high) allow identification of patterns, where higher feature values may correlate with stronger positive or negative effects on the model’s prediction. Features with a wide spread of SHAP values have a greater impact on the model’s prediction. Figure 3 shows the results of the relative importance of each variable in the whole feature set. The features contributing most to the prediction of CMD incidence are age, different types of blood pressure measures (presence of hypertension, systolic and diastolic blood pressure), and biomarkers related to immune and metabolic processes (e.g. cholines and C-reactive protein); no psychiatric variables were present in the feature importance analysis.

## Discussion

The main aim was to determine which sets of features were best able to predict onset of CMD in a sample of subjects enriched with depressive and anxiety disorders, and whether certain combinations of domains were able to increase predictive performance. Overall we found similar predictions across single domains, with a higher performance in demographics and lifestyle & somatic indicators. We found limited improvements in model performance when adding single domains to the demographics, with the best performing combination of domains was the combination of demographics, lifestyle & somatic indicators and psychiatric variables. Overall prediction (combining all domains) did not improve single-domain models. The most relevant variables in the prediction were blood pressure measures and age; no psychiatric variables showed up among the top predicting features in the full model.

The scientific literature does suggest an increased relative risk of CVD for subjects with depression. While psychiatric variables did not increase performance of the model, our feature importance analysis shows 3 (age, blood pressure and a cholesterol feature) of the 5 components of the Framingham risk score (FRS, which is an established measure for predicting 10-year risk percentage of occurrence of coronary heart disease) [4] did end up in the SHAP feature importance procedure. A meta-analysis by Rugulies [11] summarized studies using depression as a predictor for myocardial infarction or coronary death. They selected 11 cohort studies that assessed depression via self-reported measures or diagnostic interviews, finding a potential dose-response relationship between depression severity and coronary heart diseases. Meta-analyses by Farooqi [9], and Inoue [10] studied the role of depression in predicting CVD events in people with diabetes. Both found an increased risk of developing both fatal and non-fatal CVD events in those comorbid diabetes and depression versus those with diabetes alone. The differences between the current analysis and these meta-analyses are two-fold. The first difference is not all studies corrected for the same set of covariates that was present in our feature prediction set that may explain associations between depression and CVD events (e.g. physical activity, smoking and drinking habits, BMI). A second difference is the meta-analyses summarized studies estimating relative risks, which is different from prediction. Relative risk is a form of association finding, and a feature may be strongly associated with an outcome, but it might not be a good predictor for the outcome: for instance, if the feature is very rare, or if it is explained by another feature, it will not substantially add to the prediction of the outcome.

There have been some ML studies predicting CVD, however, no studies have included detailed information on depression or anxiety [43–51]. With respect to predictive performances of these ML studies, they found seemingly higher predictive performances when predicting prevalence or onset of different types of cardiometabolic diseases However, although their performance was higher (some with AUC ROCs over 0.9), a few remarks are warranted. Either sample size was very small [43–45], outcomes were strongly imbalanced [44], no significance testing was done [45–47], occurrence rather than incidence was predicted [46], mortality was predicted in an already sick and hospitalised population [48], or the source of the data was questionable having extreme outliers with (e.g. subjects with a bodyweight of 10 kg) [49] or the ratio sensitivity/specificity was very high [50].

There might be several reasons for our model’s sub-optimal performance. Firstly, with a low CMD incidence of 24%, ML classifiers struggled to find patterns. Balancing techniques create pseudo-date to artificially increase the minority class, potentially circumventing the issue. We explored Synthetic Minority Oversampling Technique (SMOTE) [52], but it did not increase performance. Some even argue it may not significantly improve performance and could even hinder it [53]. The relatively poor performances of our classifiers could thus be explained by the incidence percentage, and there is no straight-forward way of circumventing this issue. A second reason could be attributed to how the outcome variable was defined. A clinician’s diagnosis is preferable for cardio-metabolic diseases, but NESDA lacked this data; we combined (i) medication information, (ii) biological measurements and (iii) self-reported disease. Although the overlap in information form these three different sources suggests our outcome variables are robust, misclassification may still have occurred. Thirdly, it is important to note that the data had a relatively wide age variance (18–65 years at baseline), whereas for prediction purposes a more uniform or stratified sample might be better able to predict CMD. Research with higher performance generally confirms this trend as they often employ stratified or pre-selected samples [46, 48, 54]. Other potential explanations are the limited sample size of 2,071 and that it is hard to predict CMD up to 9 years in advance. Additional to limited performance, we note that the analysis was longitudinal in a restricted sense: although the outcome assessment was longitudinal, the analysis did not incorporate time to onset or time-related events. This is of importance especially for psychopathology that may have a recurrent pattern. Our prediction model included the baseline assessment of depression and anxiety and could not consider potential recurrent episodes potentially closer in time with the onset of CMD; this may have reduced the prediction accuracy of psychopathology variables. Implementing machine learning analyses catered for longitudinal analyses may further enhance predictions and interpretability.

Concluding, we can state that although it is known that depression increases risk of CMD, ML classifiers employed in this analysis were not able to capture the set of relevant features to predict the onset of cardiometabolic diseases in a sample of participants enriched with depression and anxiety disorders. The model performance was reasonable given the type of data and the sample set. We suggest stratifying the dataset further in future research, but only in the case that a higher sample size can be assured, and either using clinical knowledge to make stronger selections on predictors, or develop stronger ML classifier methods to make these selections.

## Electronic supplementary material

Below is the link to the electronic supplementary material.

Supplementary Material 1

Supplementary Material 2

Supplementary Material 3

## Data Availability

The data used to support the findings of this study are available upon request from NESDA, Amsterdam: nesda@amsterdamumc.nl. Information on how to request the study data, including the data sharing policy, can be found at https://www.nesda.nl.
