# Supplementary material for: Predicting incident cardio-metabolic disease among persons with and without depressive and anxiety disorders: a machine learning approach
Source: Soc Psychiatry Psychiatr Epidemiol. 2025 Feb 18;60(6):1457–66. doi: 10.1007/s00127-025-02857-9 (PMC12162734; doi:10.1007/s00127-025-02857-9)
Supplement: Supplementary file 2 — Supplementary Material 2 [file 127_2025_2857_MOESM2_ESM.docx]

Supplemental materials for Predicting incident cardio-metabolic disease among persons with and without depressive and anxiety disorders: a machine learning approach

**Author list:** Arja O. Rydin^[[1]](#footnote-1),^^[[2]](#footnote-2)^, George Aalbers^1,2^, Wessel A. van Eeden^[[3]](#footnote-3)^, Femke Lamers^1,2^, Yuri Milaneschi^1,2,^^[[4]](#footnote-4)^ , Brenda W.J.H. Penninx^1,2,^4

**Correspondence address:** E-mail: [a.o.rydin@amsterdamumc.nl](mailto:a.o.rydin@amsterdamumc.nl)

**ORCID digits:** 0000-0002-5525-3350, 0000-0002-7140-1536, 0000-0002-8913-0615, 0000-0003-4344-5766, 0000-0002-3697-6617, 0000-0001-7779-9672

**Journal:** Social Psychiatry and Psychiatric Epidemiology

# Supplemental methods

This work presents the results of the randomised search of the 4 classifiers: Logistic regression (LR), Support Vector Machine (SVM), Random Forest (RF), and XGBoost (XGB), performed on the 5 datasets: demographic (demo), lifestyle and somatic indicators (lifestyle), biological (bio), and psychiatric (psych). We present the 20 resulting models by showing their confusion matrix, evaluation metrics, and ROC curve. The document is divided by classifier, each section contains the results of a classifer performed on the 5 datasets.

# Logistic regression
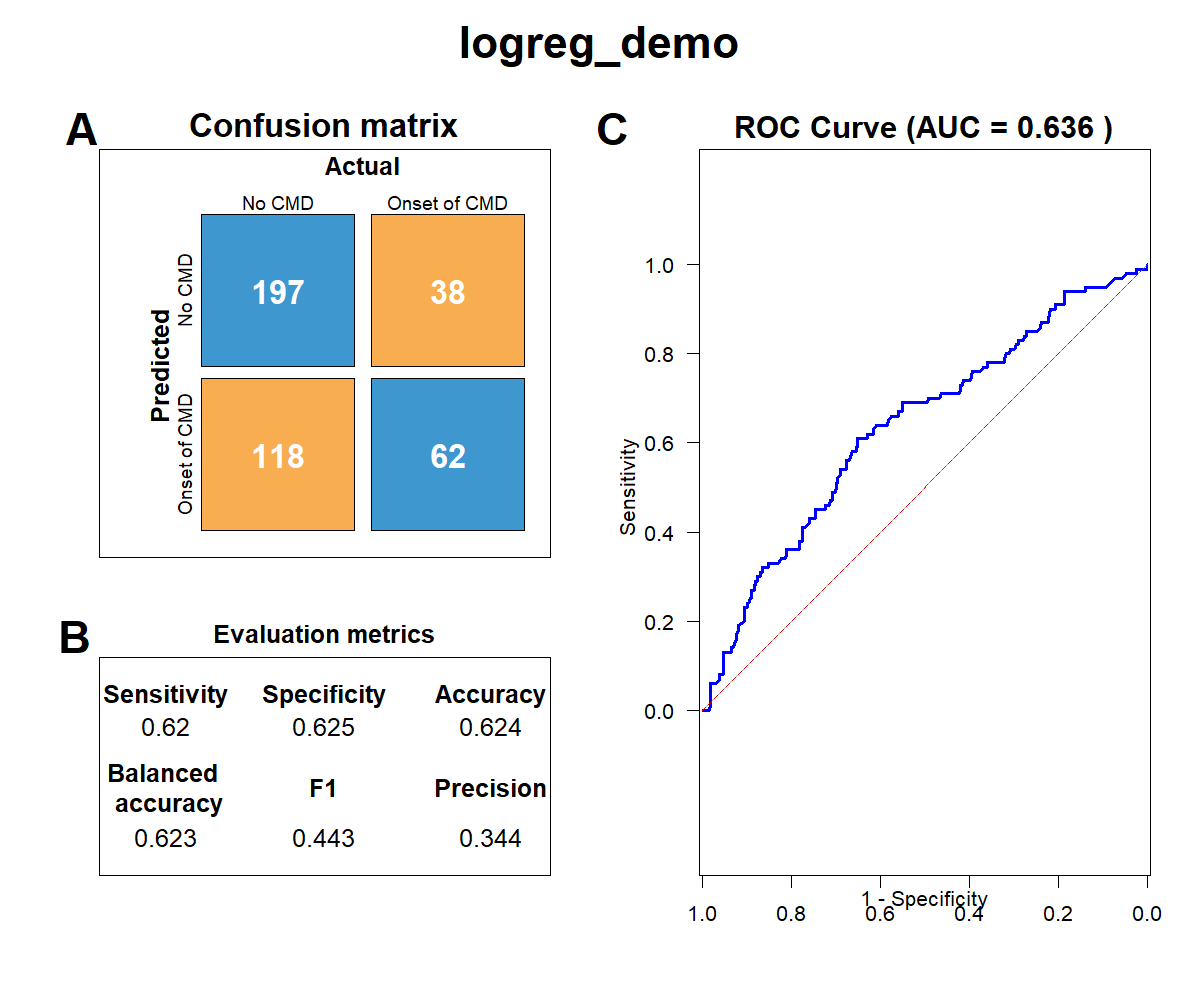


Figure S1 LR on demographic variables.

#
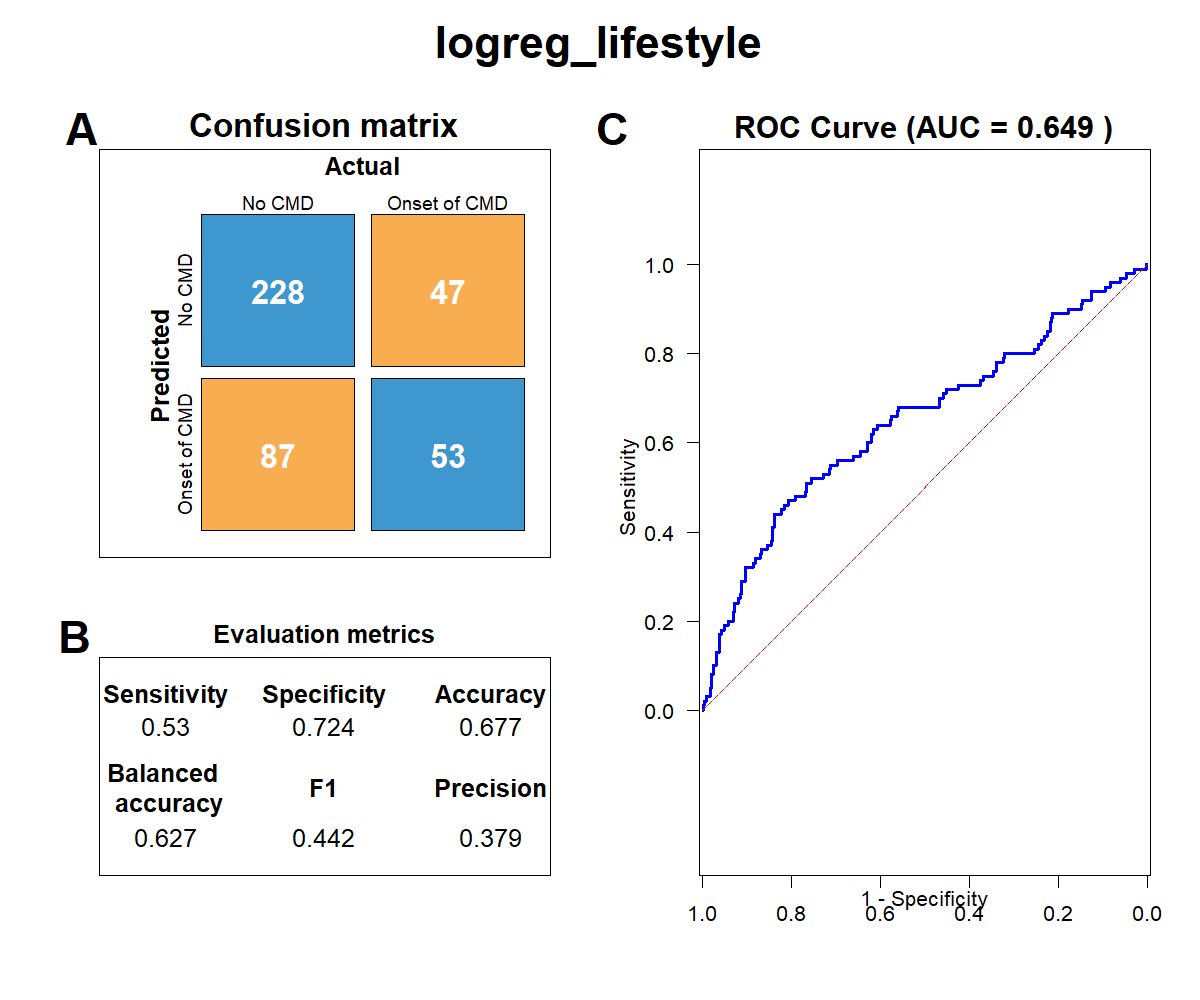


Figure S2 LR on lifestyle and somatic indicators.

#
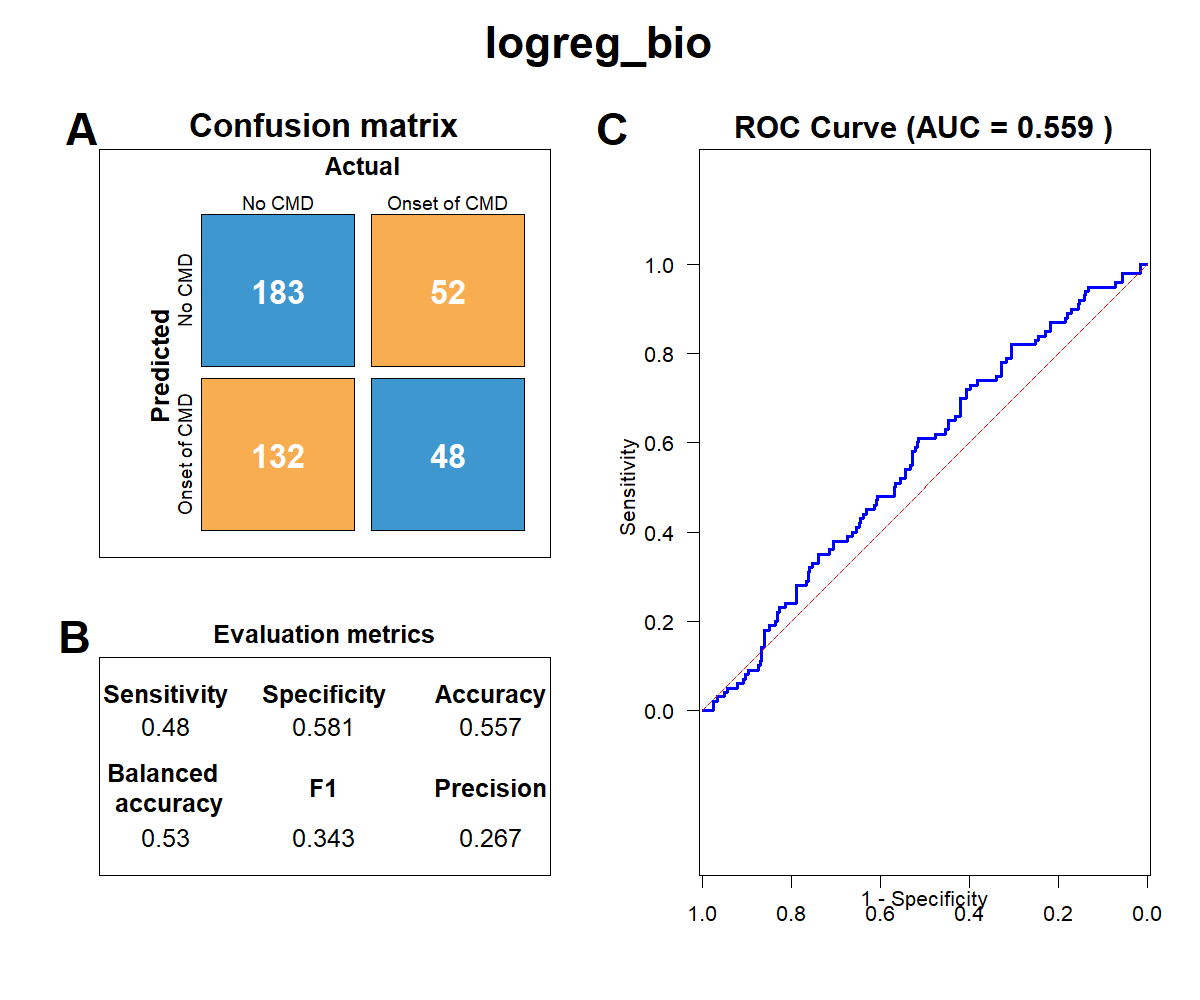


Figure S3 LR on biological variables.

#
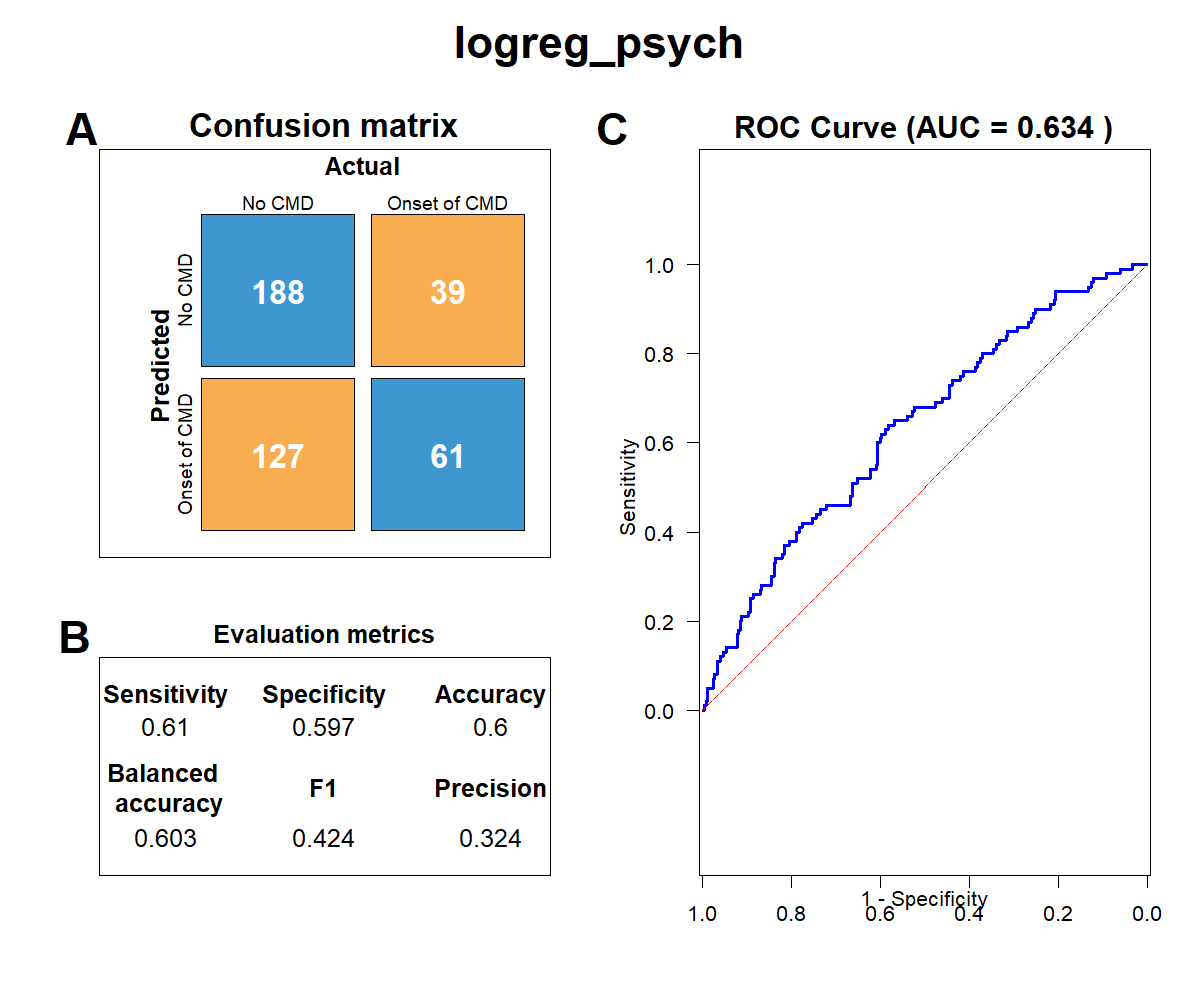


Figure S4 LR on psychiaric variables.


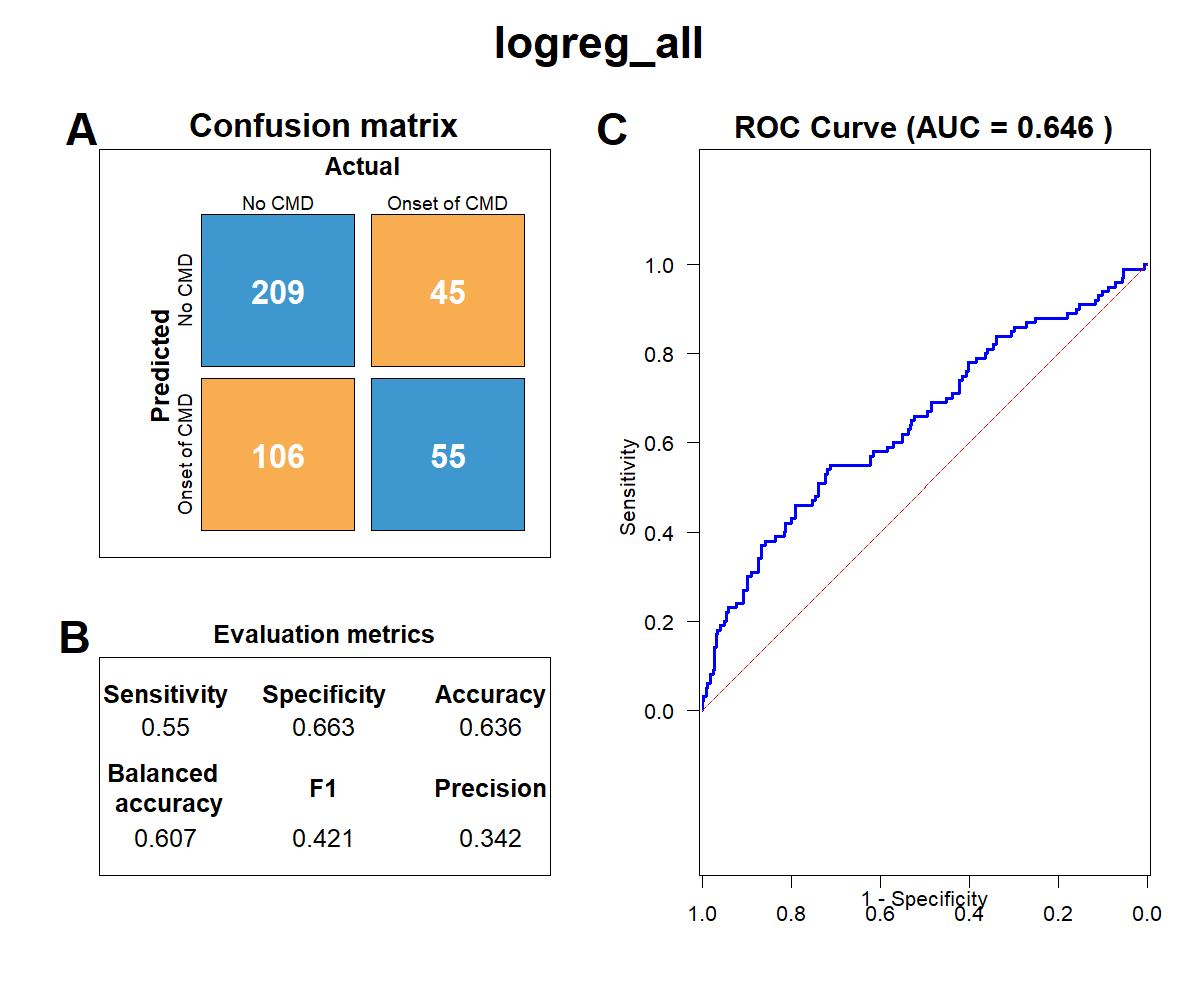


Figure S5 LR on full dataset.

# Support Vector Machine
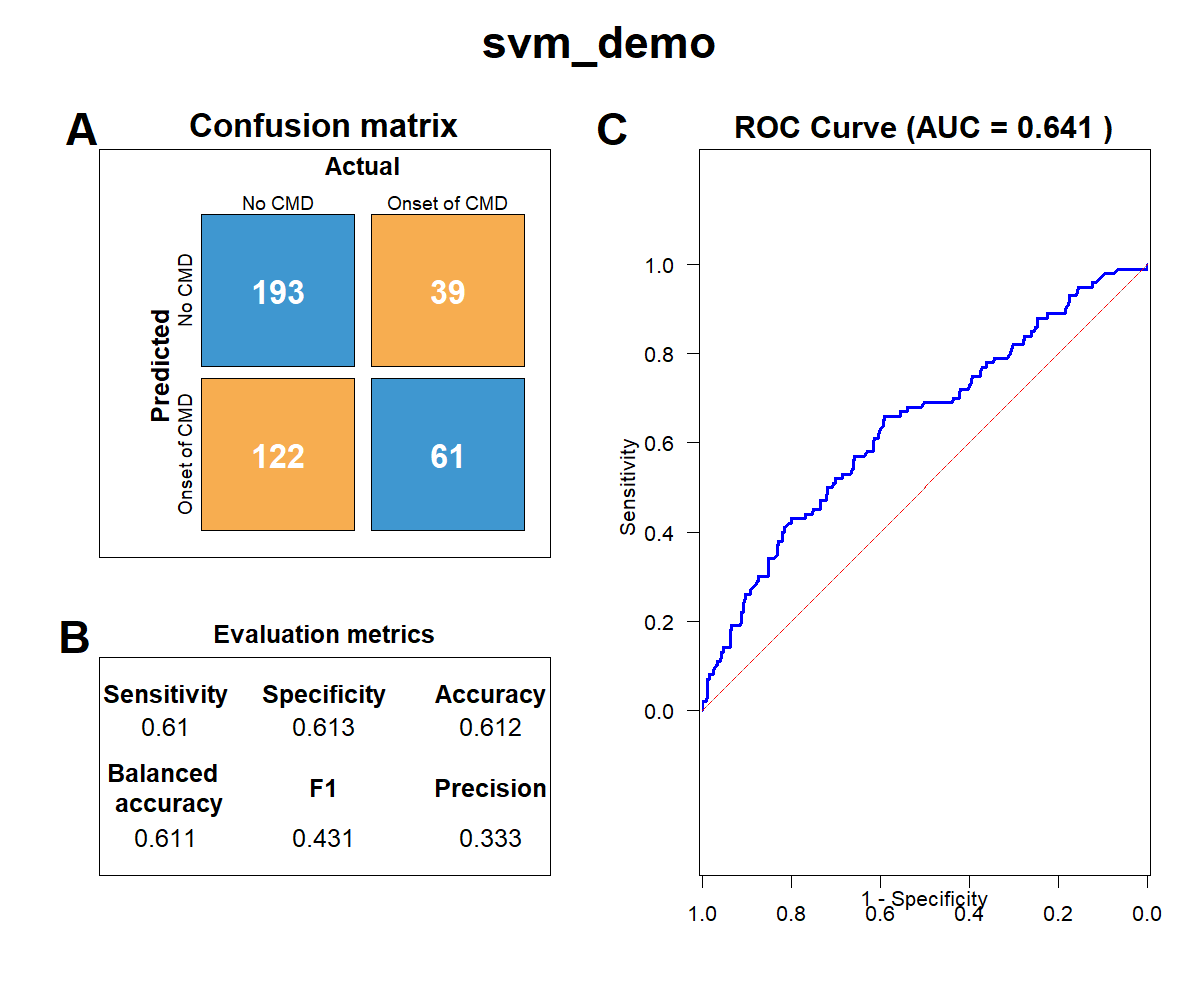


Figure S6 SVM on demographic variables.

#
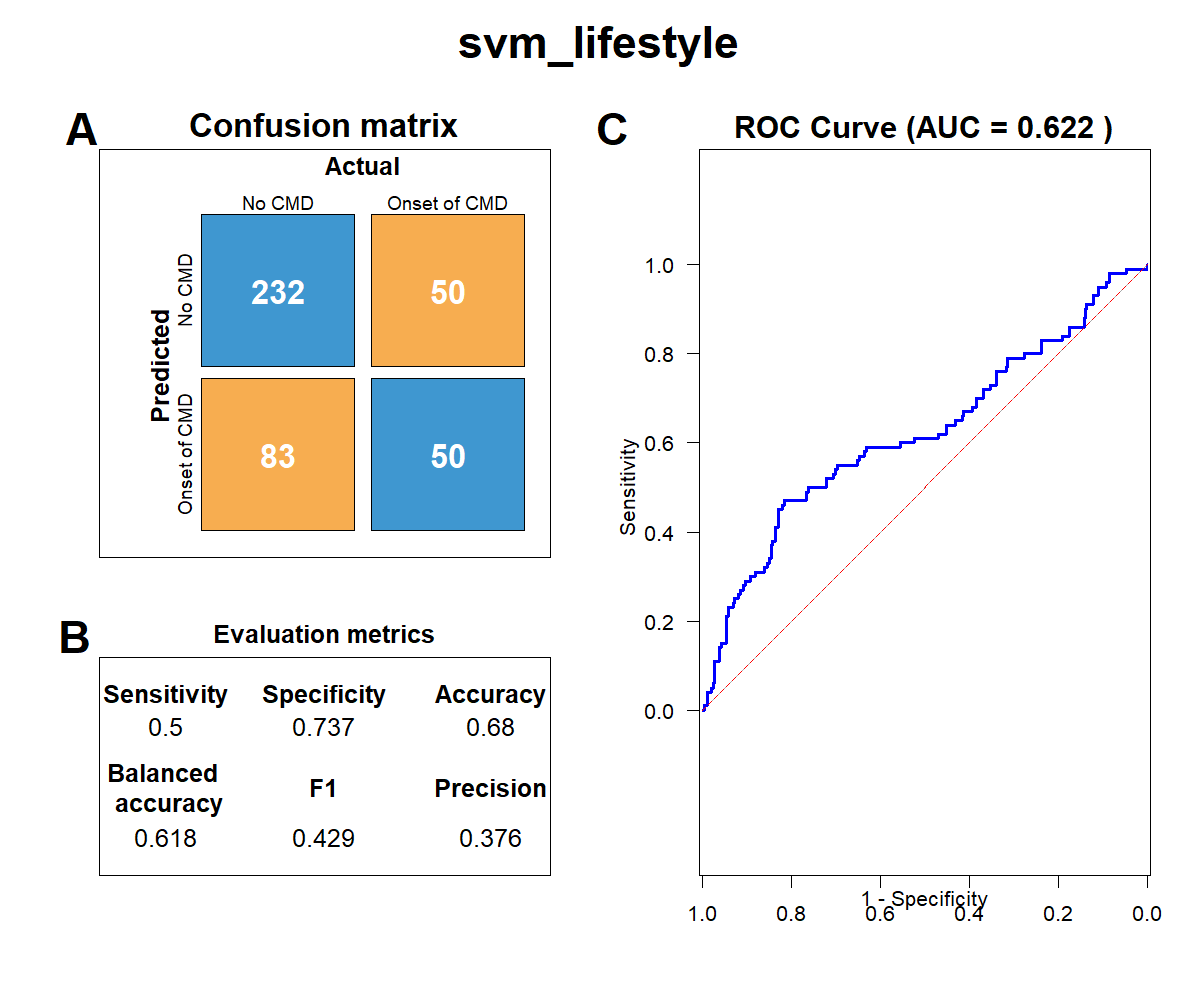


Figure S7 SVM on lifestyle and somatic indicators.

#
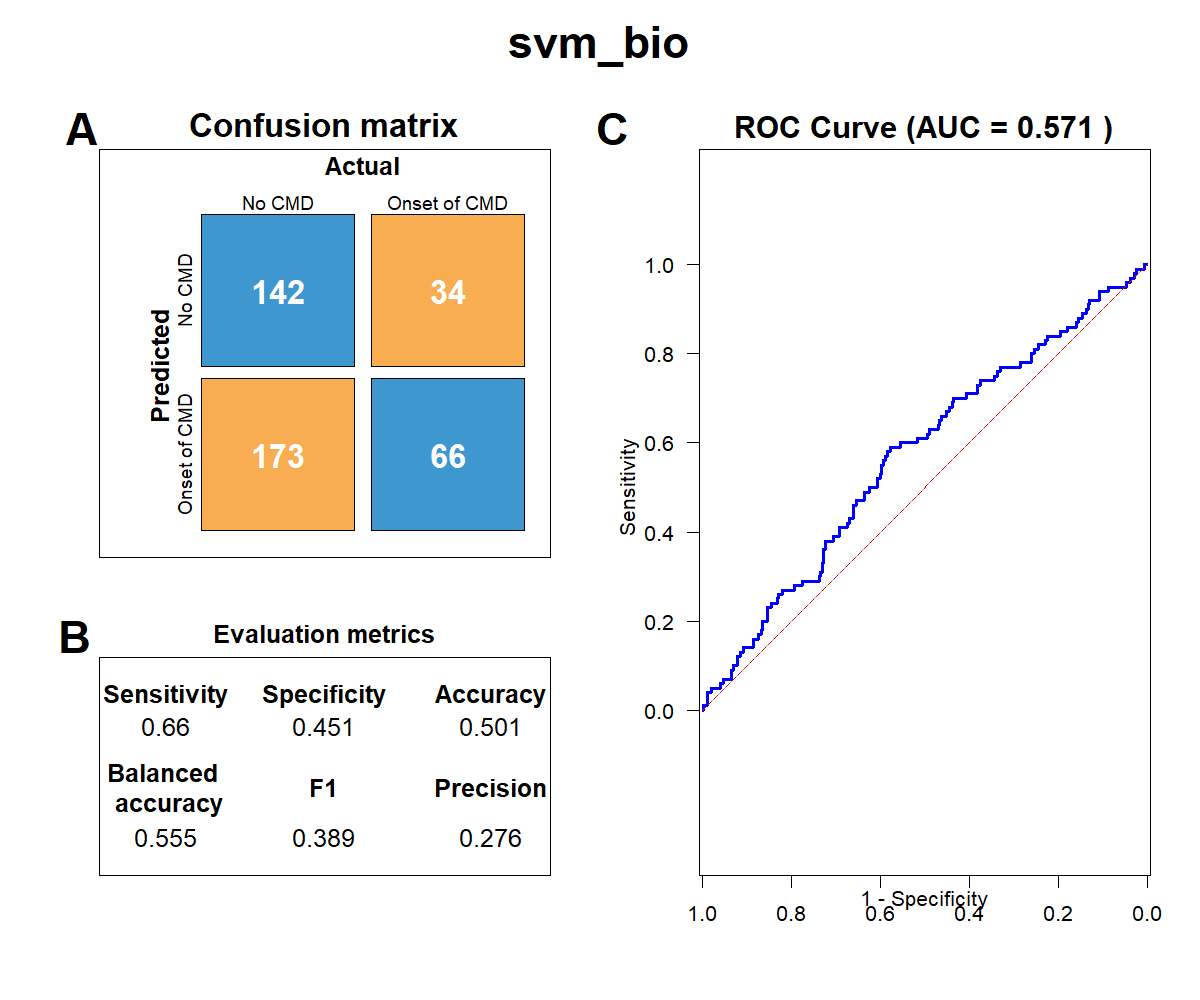


Figure S8 SVM on biological variables.

#
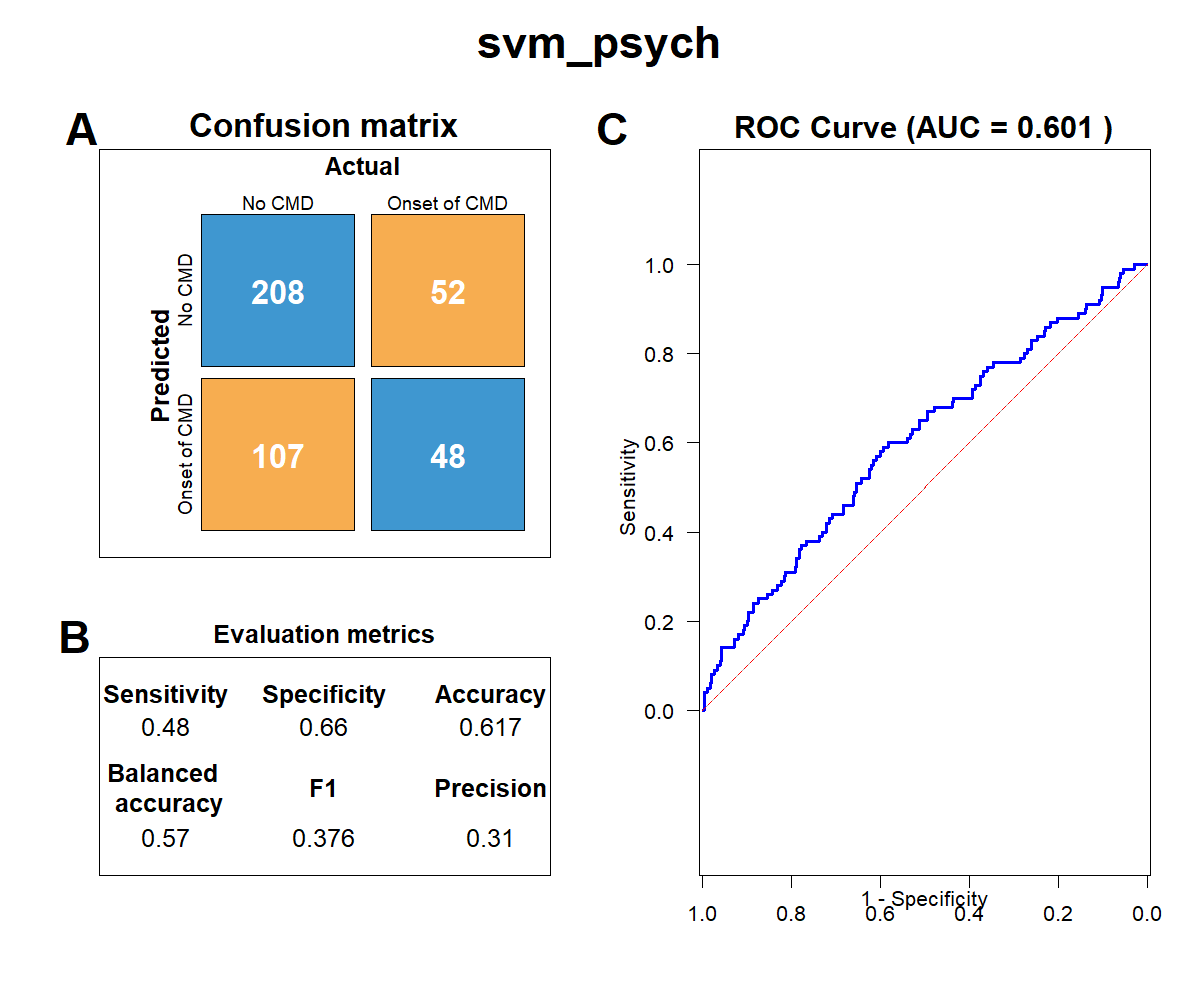


Figure S9 SVM on psyciatric variables.

#
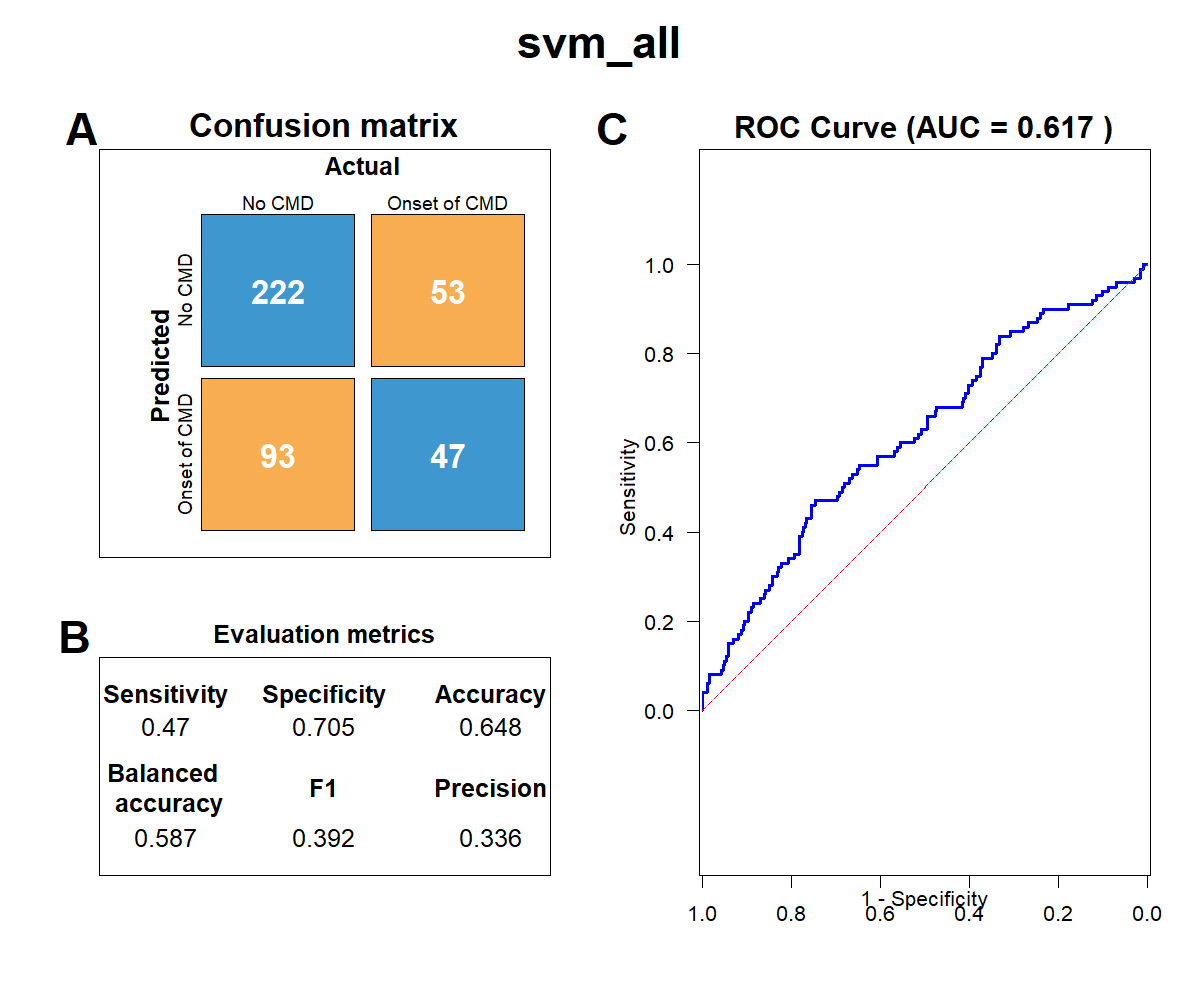


Figure S10 SVM on full dataset.

# Random Forest
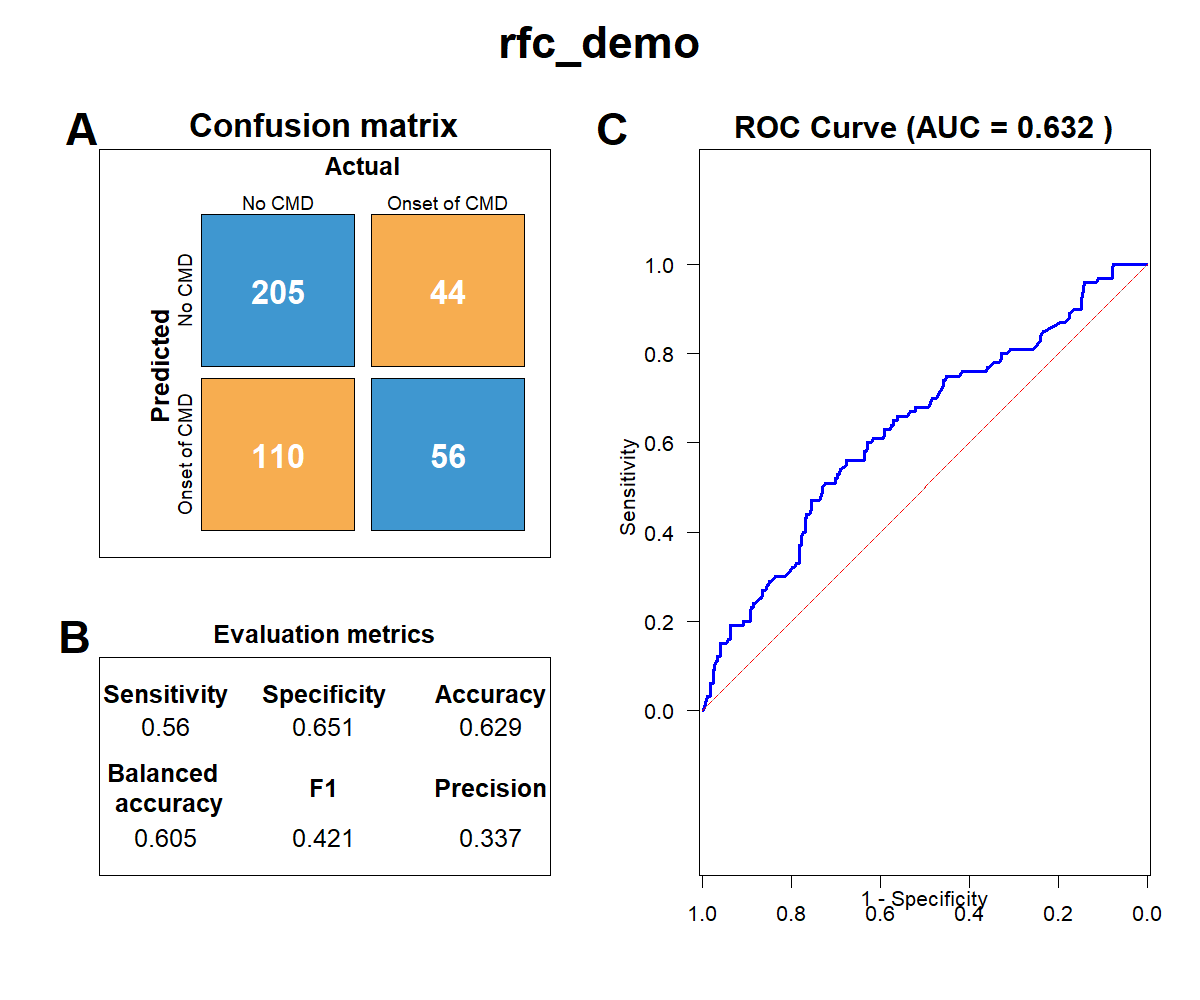


Figure S11 RF on demographic variables.

#
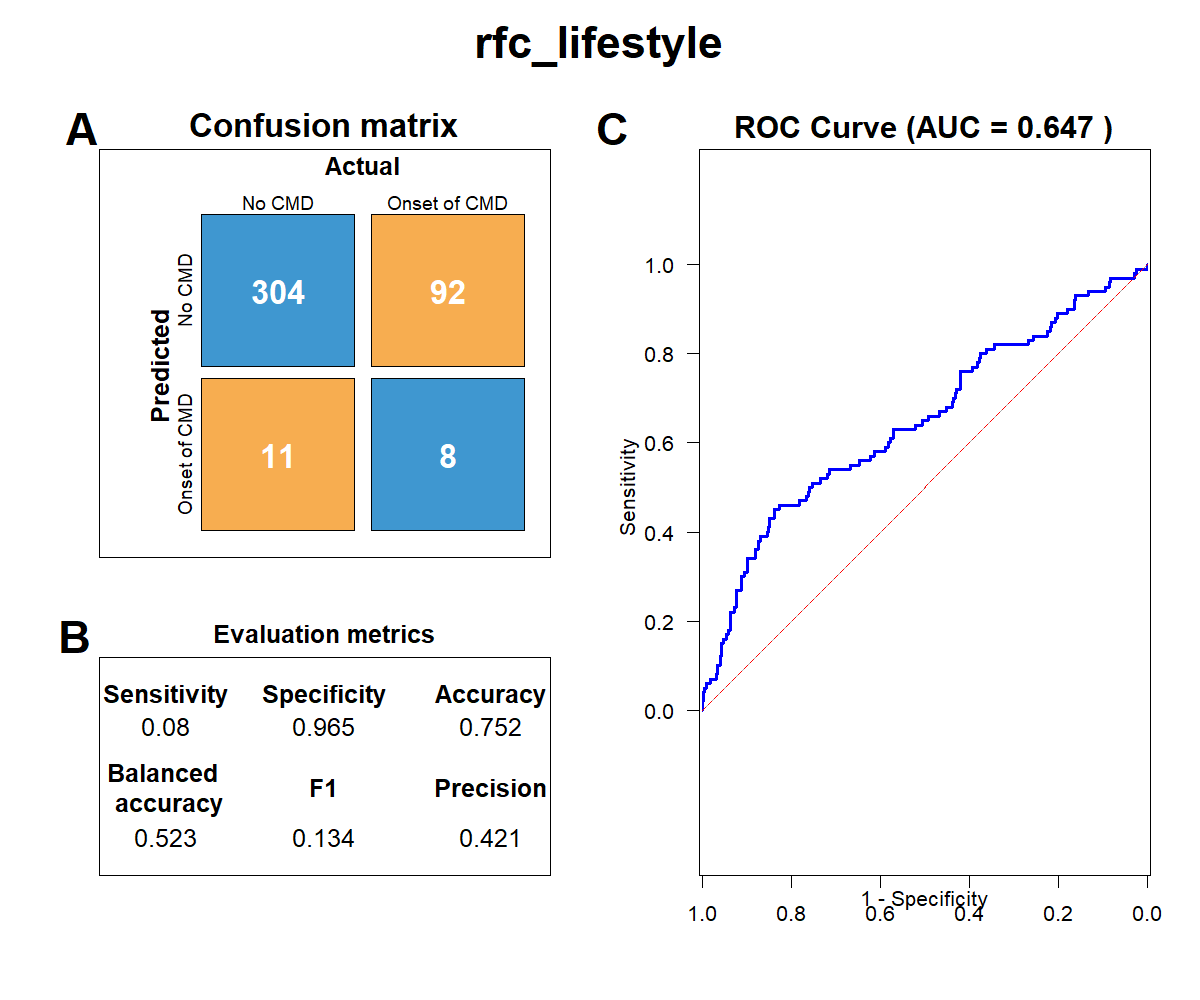


Figure S12 RF on lifestyle and somatic indicators.


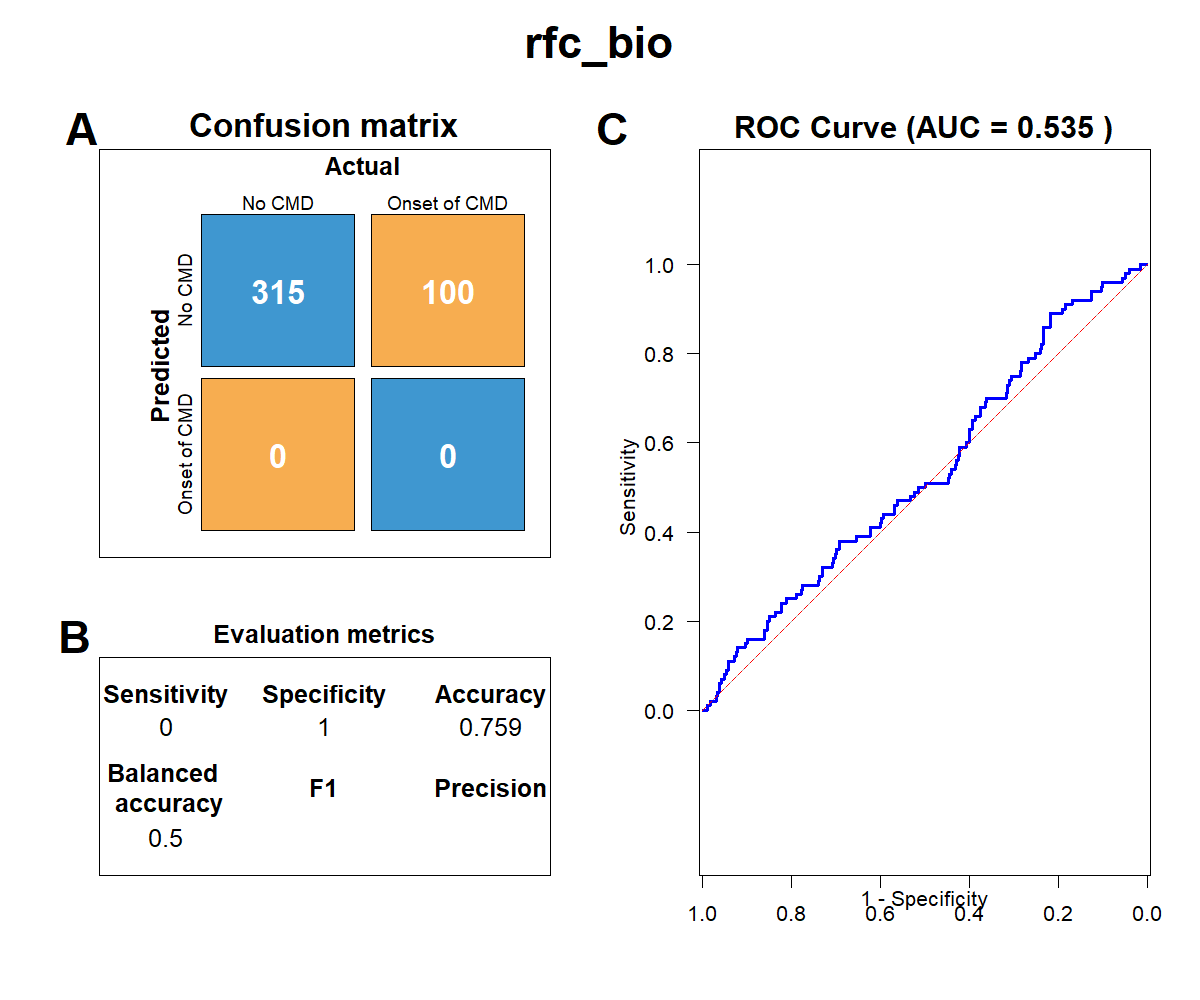


Figure S13 RF on biological variables.


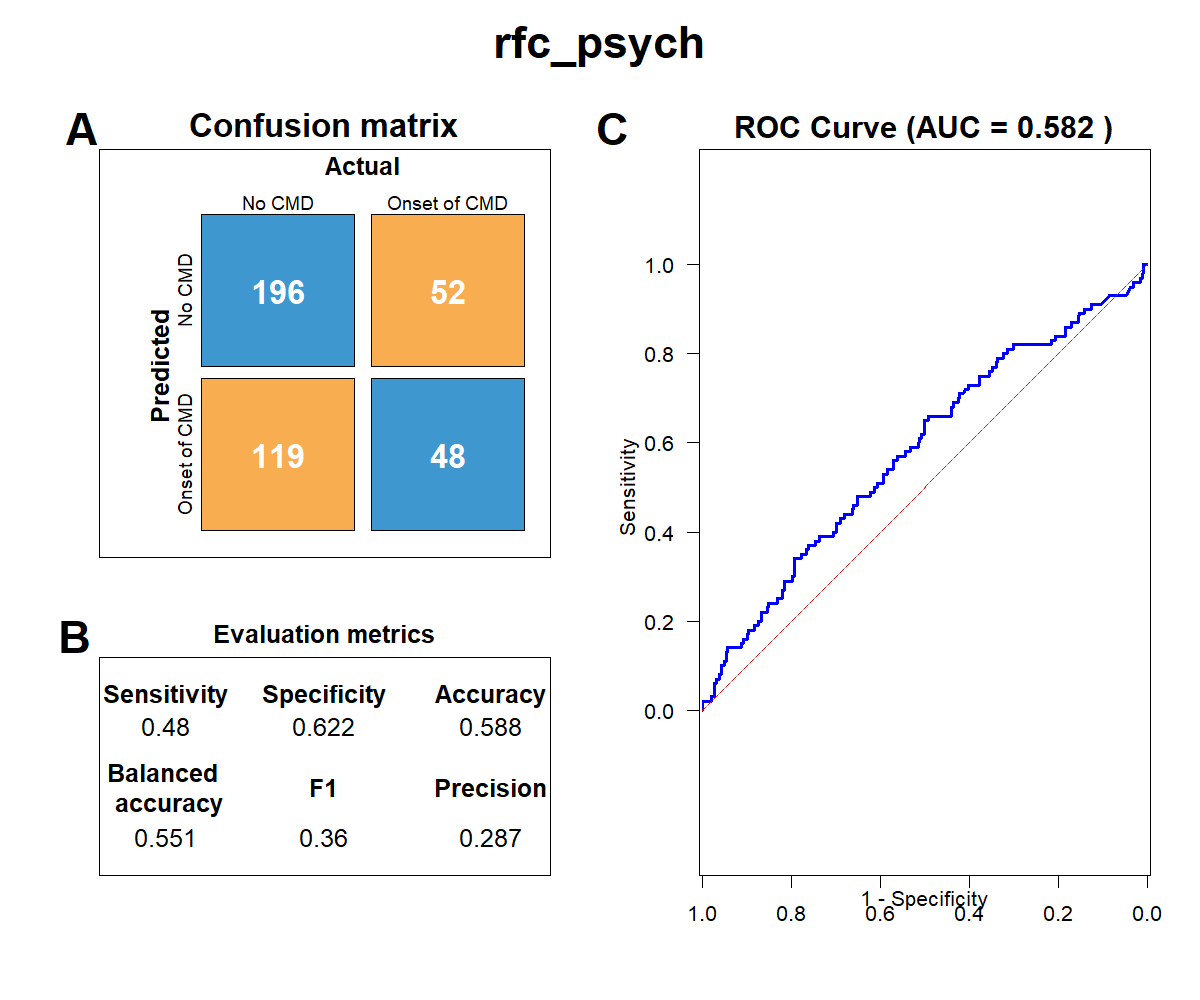


Figure S14 RF on pyschiatric variables.


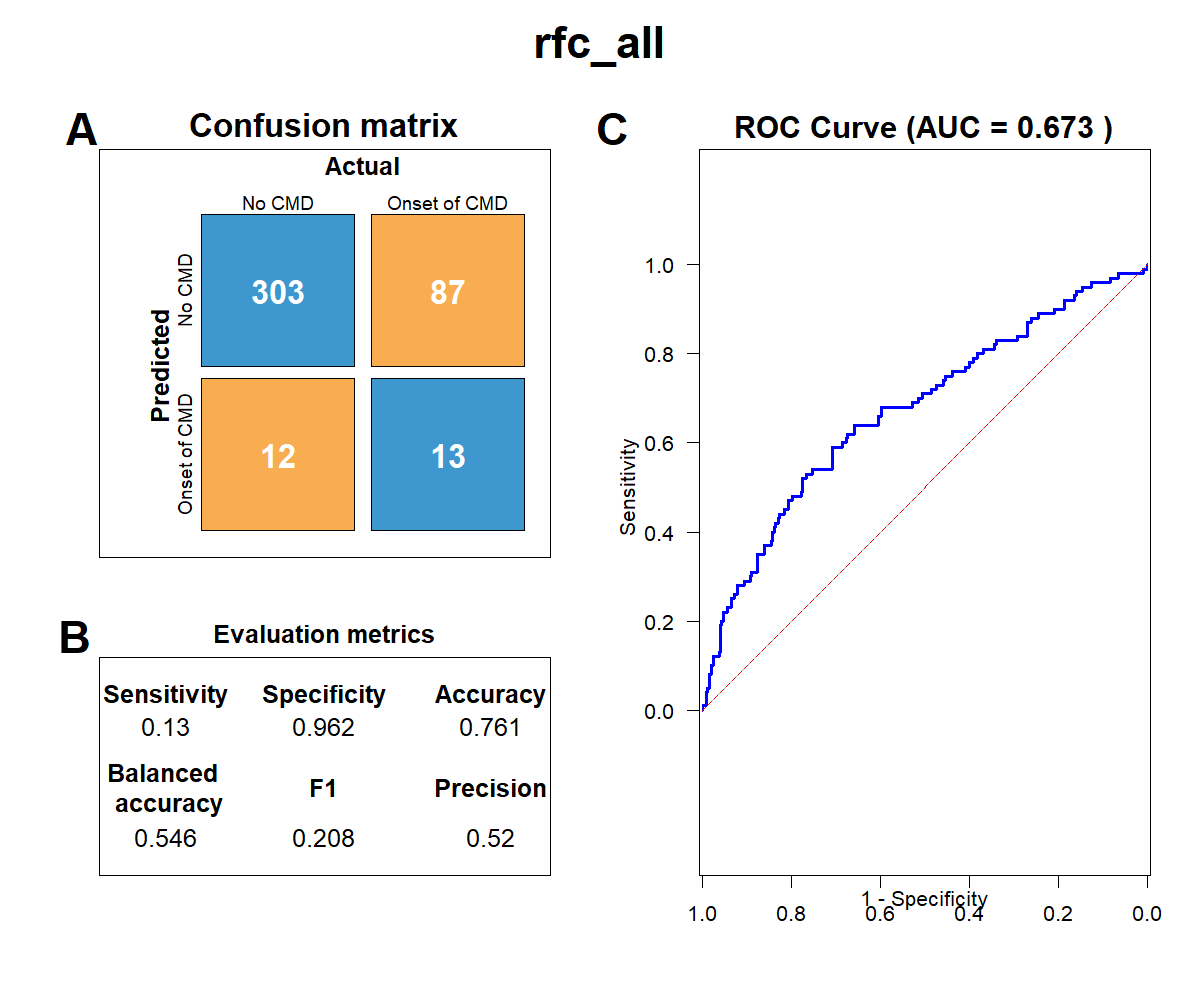


Figure S15 RF on full dataset.

# XGBoost
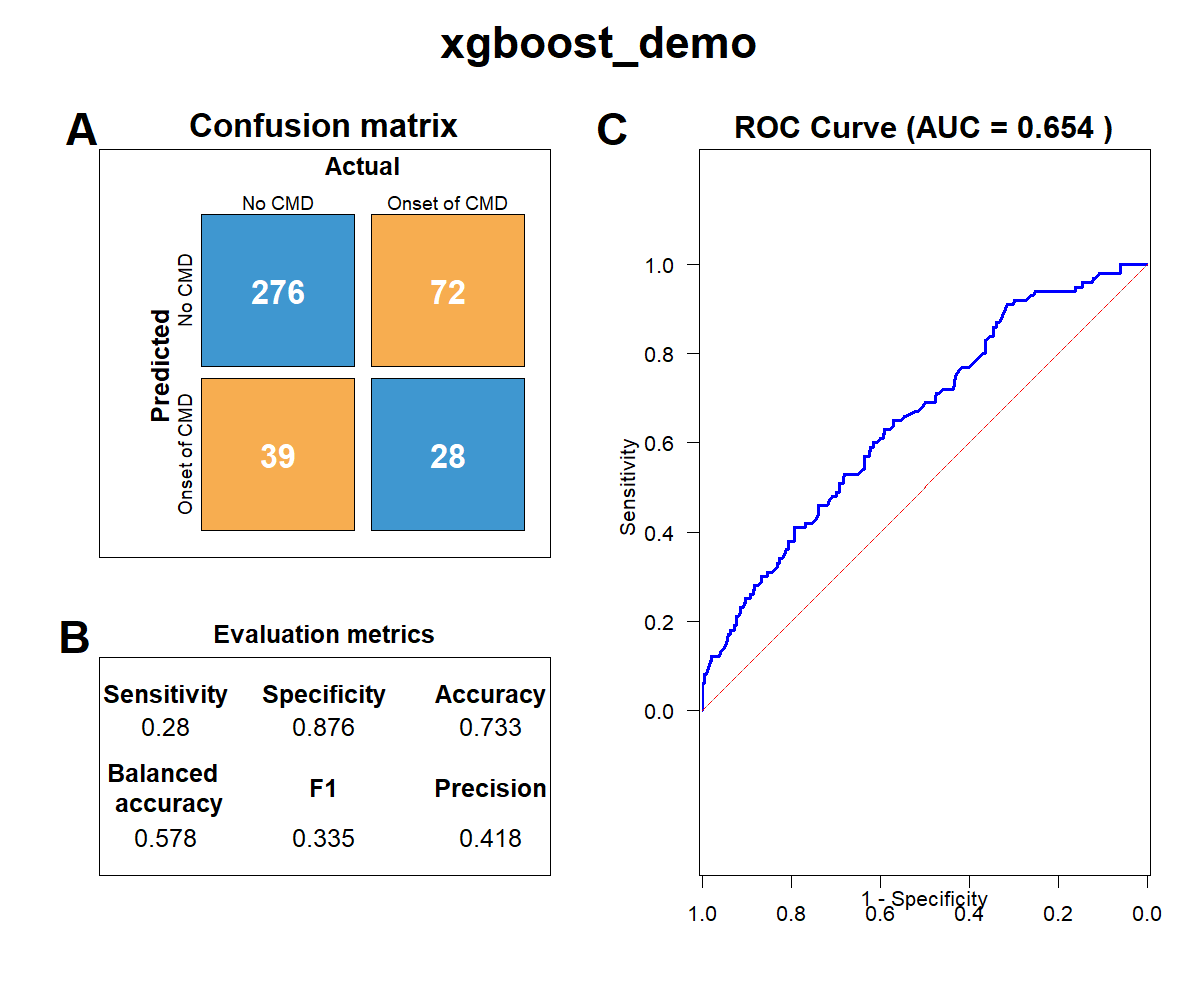


Figure S16 XGB on demographic variables.

#
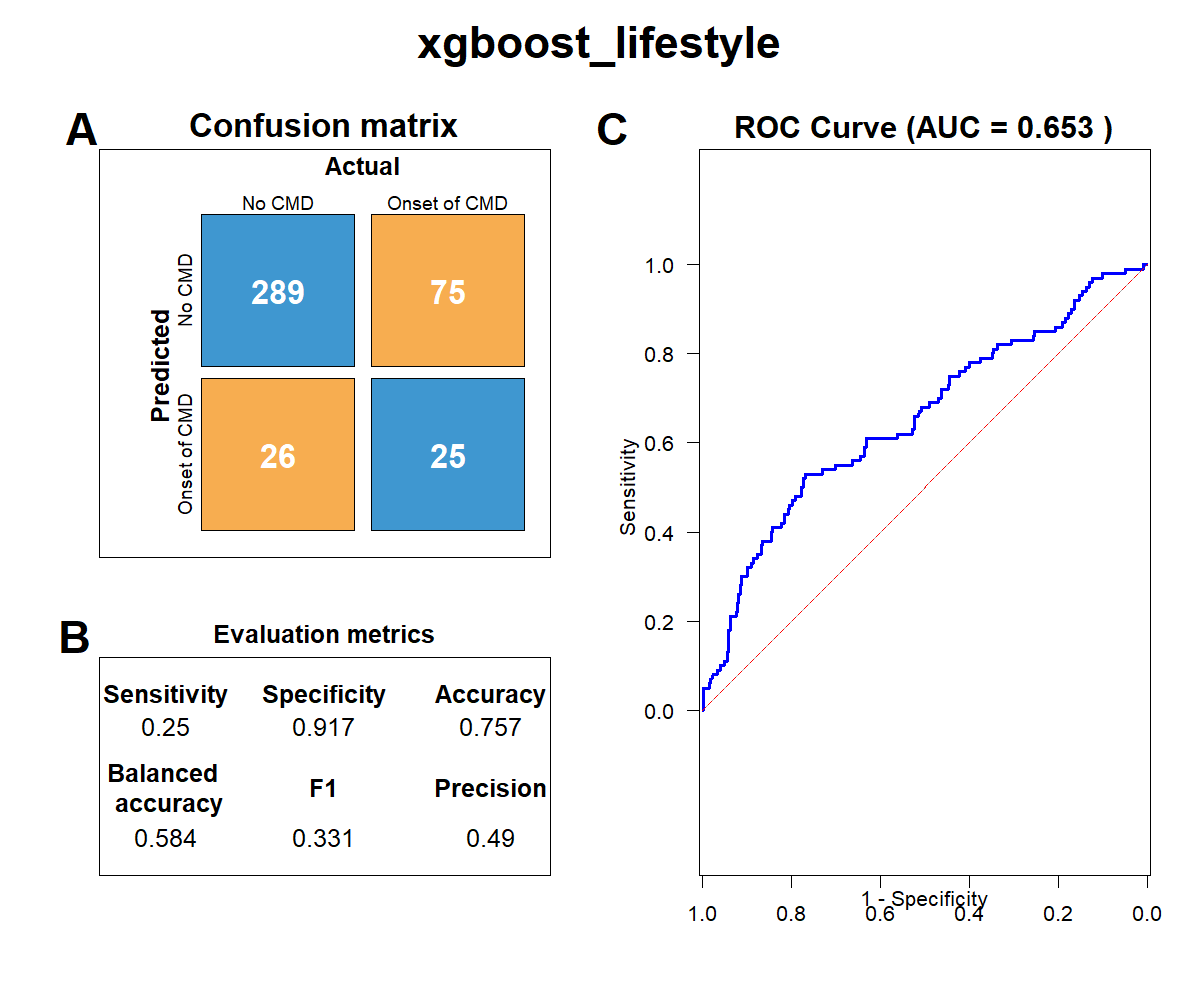


Figure S17 XGB on lifestyle and somatic indicators.

#
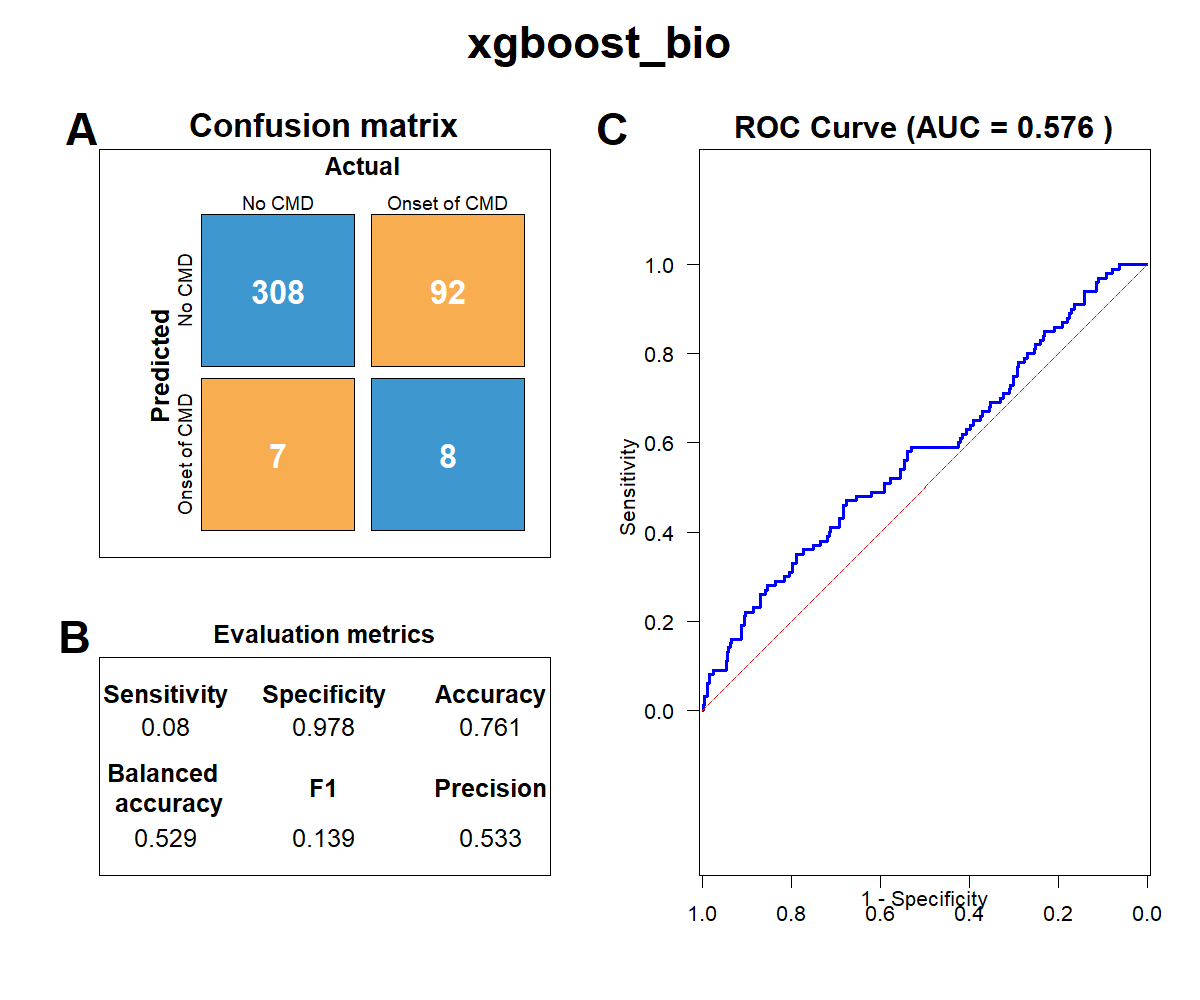


Figure S18 XGB on biological variables.


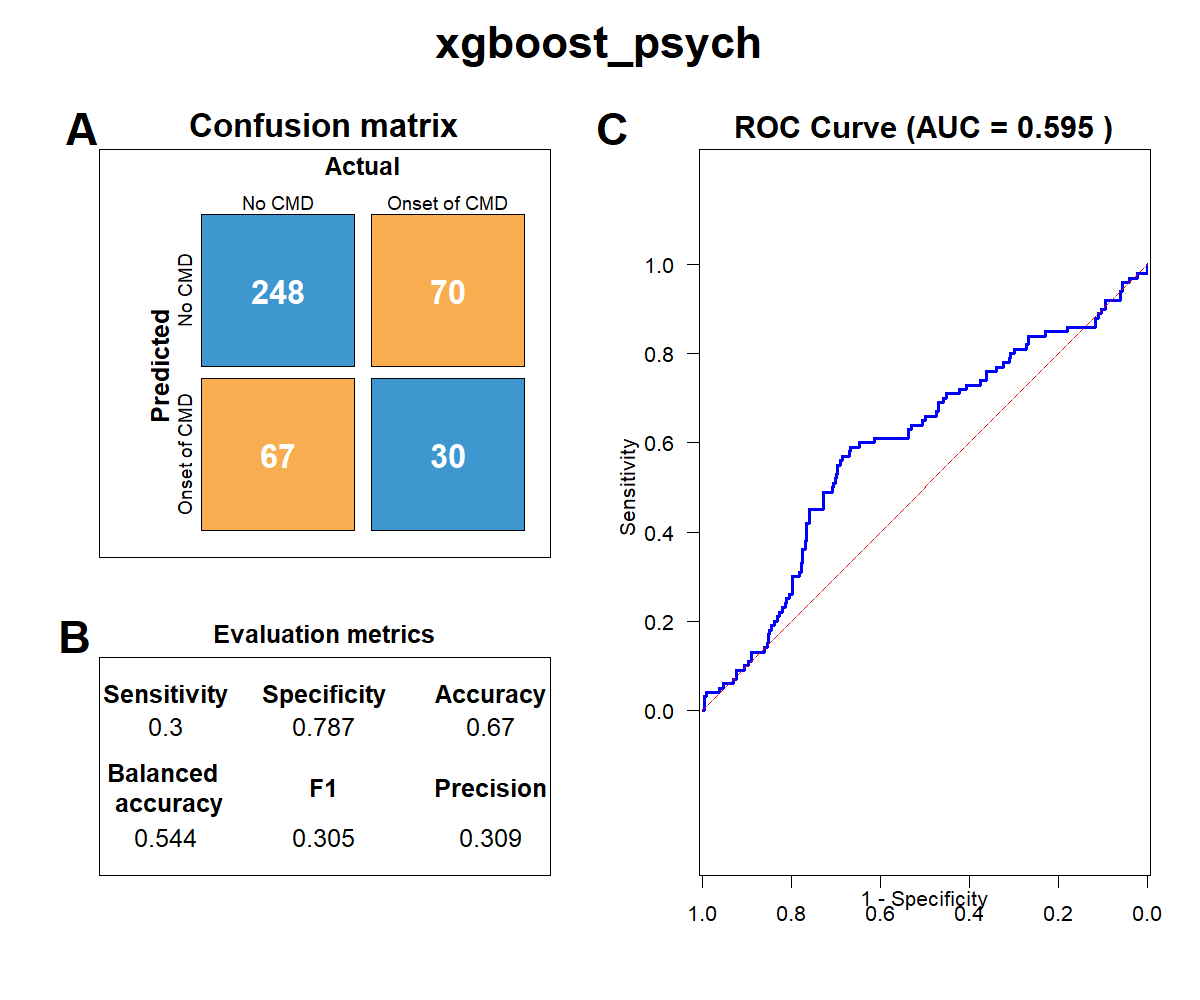


Figure S19 XGB on psychiatric variables


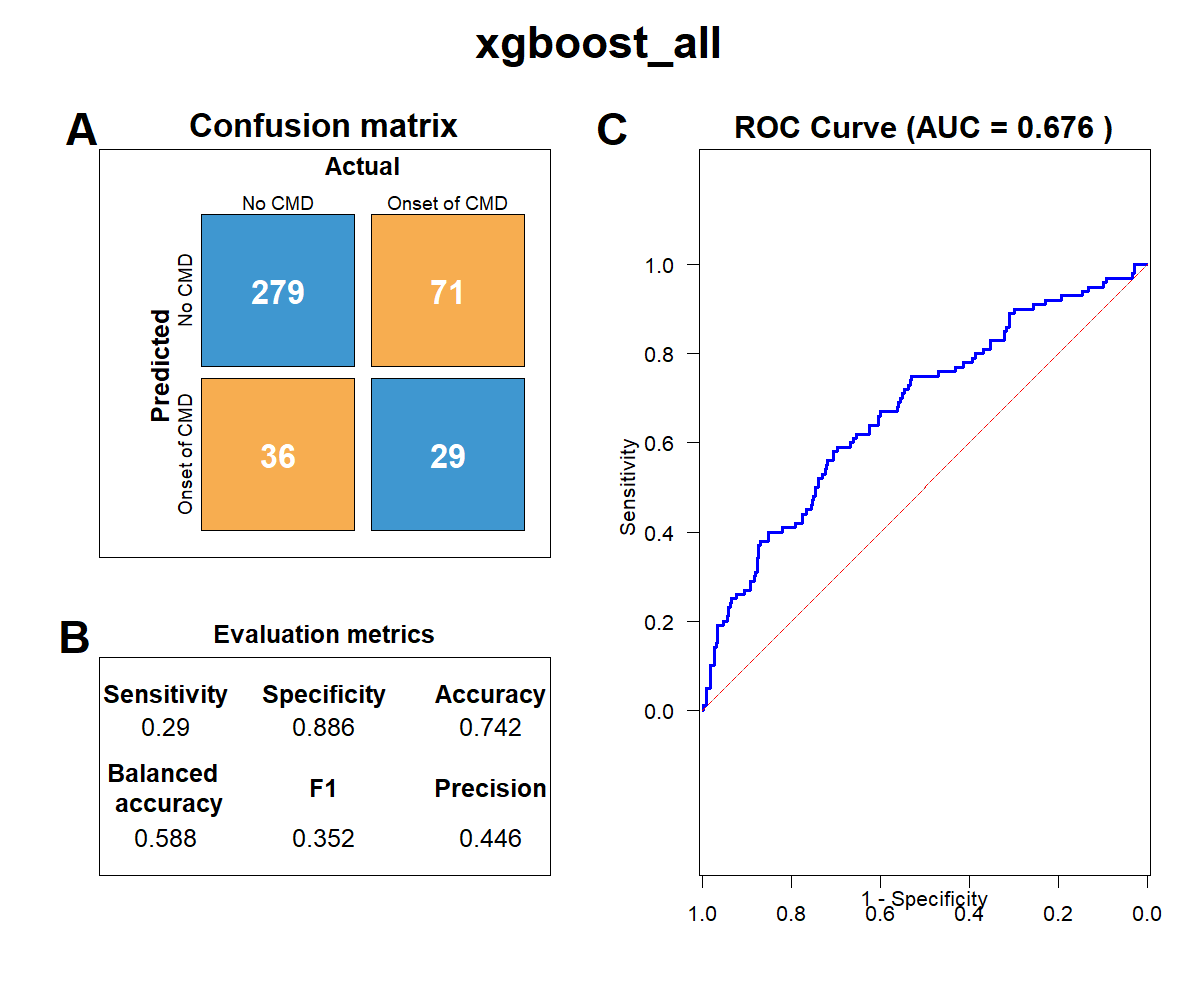


Figure S20 XGB on full dataset.

1. Department of Psychiatry, Amsterdam UMC location Vrije Universiteit Amsterdam, Boelelaan 1117 Amsterdam, The Netherlands; [↑](#footnote-ref-1)
2. Amsterdam Public Health, Mental Health Program, Amsterdam, The Netherlands; [↑](#footnote-ref-2)
3. Department of Psychiatry, Leiden University Medical Centre, Leiden University, Leiden, The Netherlands [↑](#footnote-ref-3)
4. Amsterdam Neuroscience, Mood, Anxiety, Psychosis, Sleep & Stress program, Amsterdam, The Netherlands [↑](#footnote-ref-4)
